# Three Closely Related *Spodoptera* Species Similarly Affect Gene Expression and Phytohormone Levels but Differentially Induce Volatile Emissions in Maize

**DOI:** 10.1111/pce.70389

**Published:** 2026-01-19

**Authors:** Wenfeng Ye, Sara Leite Dias, Marine Mamin, Carla C. M. Arce, Ted C. J. Turlings

**Affiliations:** ^1^ Laboratory of Fundamental and Applied Research in Chemical Ecology, Institute of Biology University of Neuchâtel Neuchâtel Switzerland; ^2^ CAS Key Laboratory of Insect Developmental and Evolutionary Biology, Center for Excellence in Molecular Plant Sciences, Shanghai Institute of Plant Physiology and Ecology Chinese Academy of Sciences Shanghai China; ^3^ Metabolic Diversity Group, Department of Molecular Genetics Leibniz Institute of Plant Genetics and Crop Plant Research (IPK) Gatersleben Germany; ^4^ Laboratory of Plant‐Insect Research for Sustainable Agriculture (PIRSA), State Key Laboratory of Cotton Bio‐Breeding and Integrated Utilization, School of Life Sciences Henan University Kaifeng China; ^5^ Department of Entomology, The Pennsylvania State University University Park State College Pennsylvania USA

**Keywords:** oral secretion, phytohormones, *Spodoptera exigua*, *Spodoptera frugiperda*, *Spodoptera littoralis*, transcriptome, *Zea mays*

## Abstract

Plants can perceive specific elicitors in the oral secretions (OS) of herbivorous insects and respond by increasing their defences. Whether plants can discriminate among similar herbivorous insect species and differentially modulate their defence responses against them is largely unknown. Here, we investigated the responses of the maize transcriptome, phytohormones, and volatile emissions to the OS of three closely related *Spodoptera* caterpillars: the fall armyworm *S. frugiperda*, the beet armyworm *S. exigua*, and the cotton leafworm *S. littoralis*. Maize plants strongly increased their phytohormone levels and volatile emissions when treated with each of the OS, which was reflected in the transcription levels of genes involved in phytohormone signalling, and primary and secondary metabolism. Compared to the OS of *S. exigua* and *S. littoralis*, the secretion of the maize specialist *S. frugiperda*, elicited greater changes in the maize transcriptome but triggered considerably lower volatile emissions. Besides revealing the generality and specificity of maize responses to different lepidopteran caterpillars, the dataset provides a molecular resource for studies that aim to identify and characterise herbivore‐specific elicitors and effectors and their receptors. This information can then be used to elucidate and possibly disrupt the mechanisms that allow well‐adapted herbivorous insects to manipulate maize defences.

## Summary statement

This study provides a multi‐omics investigation into how maize perceives and responds to herbivory by caterpillars of three closely related species. By integrating transcriptomic, phytohormonal, and volatile analyses, we reveal that the underlying transcriptional and hormonal responses are largely conserved, but that the resulting volatile emissions are distinctly species‐specific. Notably, the oral secretions of the specialist *Spodoptera frugiperda* elicited a stronger transcriptional change but a weaker volatile emission. These findings advance our fundamental understanding of specificity in plant‐herbivore interactions and create a molecular resource to help identify novel herbivore‐specific elicitors. This work might further pave the way for future strategies to disrupt the manipulation by pests of plant defences and thereby enhance crop protection.

## 1 Introduction

Plants have evolved a broad spectrum of defence mechanisms to protect themselves from the constant threat by herbivores (Karban et al. 1997; Howe and Jander 2008; Mithöfer and Boland 2012; Farmer 2014). When attacked by herbivorous insects, plants perceive insect‐derived elicitors, also referred to as herbivore‐associated molecular patterns (HAMPs), via specific receptors and then activate complex signalling networks. Early signalling such as membrane potential, calcium ion flux, reactive oxygen species (ROS), mitogen‐activated protein kinase (MAPK) cascades, and phytohormone signalling pathways including jasmonic acid (JA), salicylic acid (SA), abscisic acid (ABA), and ethylene (ET) play an essential role in mediating the expression of defence‐related genes and the production of defence compounds (Erb and Reymond 2019; Schuman and Baldwin 2016; Wu and Baldwin 2010). Plants can defend themselves directly by producing toxic secondary metabolites or defensive proteins that lead to behavioural avoidance and/or reduced insect performance (Erb and Reymond 2019). In addition, plants may protect themselves indirectly by emitting herbivore‐induced plant volatiles (HIPVs) that attract the natural enemies of the herbivores (Turlings and Erb 2018). However, well‐adapted herbivores might suppress these defences by secreting specific effectors (Mutti et al. 2008; Chung et al. 2013; Jones et al. 2022).

The perception of herbivore‐derived elicitors induces characteristic defence responses in plants against herbivores (Arimura 2021; Jones et al. 2022). Thus far, several elicitors such as β‐glucosidase, fatty acid‐amino acid conjugates (FACs), inceptins, caeliferins and vitellogenins have been identified in insect oral secretions (OS) and their roles in mediating specific defence responses in plants have been extensively studied (Mattiacci et al. 1995; Alborn et al. 1997; Halitschke et al. 2001; Pohnert et al. 1999; Turlings et al. 2000; Schmelz et al. 2006; Alborn et al. 2007; Zeng et al. 2023). Much less is known about insect‐produced effectors that suppress plant defences. It has been reported that the glucose oxidases (GOX) in OS from caterpillars of beet armyworm *Spodoptera exigua* and bollworm *Helicoverpa zea* function as effectors, suppressing the expression of genes involved in terpenoid biosynthesis in barrel medic (*Medicago truncatula*) and inhibiting toxic nicotine production in tobacco (*Nicotiana tabacum*), respectively (Musser et al. 2002; Bede et al. 2006). In maize, GOX may suppress the emissions of green leaf volatiles (GLVs) but enhance the release of terpenoids (Jones et al. 2023). The OS of the cabbage white butterfly *Pieris brassicae* and the cotton leafworm *Spodoptera littoralis* suppress wound‐induced responses in *Arabidopsis thaliana* (Consales et al. 2012), while OS of the fall armyworm *Spodoptera frugiperda* was found to suppress the volatile emission in maize plants (De Lange et al. 2020), suggesting the presence of potential effectors in the OS of this lepidopteran herbivore besides elicitors. The mechanisms of how effectors, together with elicitors, modulate defence responses in plants, are still largely unknown.

Maize (*Zea mays* L.) is one of the most important staple crops grown worldwide. Benzoxazinoids (BXs), a class of indole‐derived secondary metabolites, play a critical role in defending maize against herbivorous insects (Frey et al. 2009). Their biosynthetic pathway along with the enzymes involved in their formation are well studied (Frey et al. 2009; Tzin et al. 2017; Handrick et al. 2016). The most abundant BX in undamaged leaves of many maize lines is 2,4‐dihydroxy‐7‐methoxy‐1,4‐benzoxazin‐3‐one β‐d‐glucopyranose (DIMBOA‐Glc) (Glauser et al. 2011; Meihls et al. 2013). In response to caterpillar feeding, its derivative 2‐hydroxy‐4,7‐dimethoxy‐1,4‐benzoxazin‐3‐one β‐d‐glucopyranose (HDMBOA‐Glc) accumulates in a highly localised manner and becomes the main BX metabolite (Glauser et al. 2011; Marti et al. 2013; Maag et al. 2016). After tissue damage by chewing herbivores, DIMBOA‐Glc and HDMBOA‐Glc are hydrolysed by plant‐derived β‐glucosidases, leading to the release of 2,4‐dihydroxy‐7‐methoxy‐1,4‐benzoxazin‐3‐one (DIMBOA) and 2‐hydroxy‐4,7‐dimethoxy‐1,4‐benzoxazin‐3‐one (HDMBOA), respectively (Oikawa et al. 1999; Glauser et al. 2011). DIMBOA and HDMBOA are deterrents and toxic to various lepidopteran herbivores, including *S. exigua* and *S. littoralis* (Rostás 2007; Glauser et al. 2011). Moreover, *S. exigua* and *S. littoralis* caterpillars perform better on *BX*‐deficient mutant lines than wild‐type maize plants (Maag et al. 2016; Tzin et al. 2017). However, well‐adapted maize pests such as *S. frugiperda* can tolerate and detoxify maize BXs (Glauser et al. 2011; Israni et al. 2020) and even utilise BXs as foraging cues (Köhler et al. 2015).

In addition to non‐volatile defensive metabolites, insect‐damaged maize plants also emit a complex blend of volatile organic compounds (VOCs) that can attract natural enemies of the herbivores (Rasmann et al. 2005; Turlings et al. 1990; Tamiru et al. 2011; Guo et al. 2019), transmit stress signals within and between plants (Engelberth et al. 2004; Ton et al. 2007; Erb et al. 2015) and repel herbivores or inhibit their performance (Bernklau et al. 2016; Veyrat et al. 2016). Among the HIPVs, terpenoids play a critical role in plant indirect defence and their production is mainly regulated by the expression of genes of the terpene synthase (TPS) family. A wide variety of TPSs have been identified and characterised in maize (Schnee et al. 2002; Schnee et al. 2006; Köllner et al. 2008; Richter et al. 2016; Block et al. 2019). Terpenes released by caterpillar‐damaged maize appears mostly localised (Köllner et al. 2013, but see Turlings and Tumlinson 1992) and can be herbivore‐specific and dependent on FACs content in caterpillar OS (Sawada et al. 2006; Ling et al. 2021).

The fall armyworm *Spodoptera frugiperda* (J. E. Smith), the beet armyworm *Spodoptera exigua* (Hübner), and the cotton leafworm *Spodoptera littoralis* (Boisduval) are three closely related *Spodoptera* species (Lepidoptera: Noctuidae) widely distributed across the globe and are important polyphagous agricultural pests destructive to a broad range of host plants, including maize (CABI, https://www.cabi.org/isc/). The latter two species originate from Africa and Southeast Asia, respectively, while *S. frugiperda*, native to tropical and subtropical regions of the Americas (Nagoshi et al. 2012; Early et al. 2018), has a close evolutionary history with maize which originated in Mexico (Matsuoka et al. 2002). All three lepidopteran species are generalists but *S. frugiperda* has a strong preference for plants of the Poaceae family such as maize (Sparks 1979). *Spodoptera frugiperda* is an exceedingly devastating pest of maize, and its ability to tolerate and manipulate the defences of this crop may be the main reason for its pest status (Glauser et al. 2011; Israni et al. 2020; De Lange et al. 2020). However, how *S. frugiperda* modulates maize plant defences via its OS is still largely unknown.

Although there is considerable information about bioactive compounds in the OS of lepidopteran insect and their role in regulating phytohormones and plant volatile emissions (Delphia et al. 2006; Schmelz et al. 2009; Acevedo et al. 2019; Jones et al. 2022; De Lange et al. 2020; Ling et al. 2021), only few studies have integrated transcriptome, phytohormone, and volatile data to compare defence responses to different herbivores. Here we did this for different *Spodoptera* species at multiple levels. As a first step towards elucidating the mechanisms by which different *Spodoptera* caterpillars affect maize plant defences, we characterised changes in transcriptome, phytohormones and volatile emissions in maize plants in response to elicitation with the OS from *S. frugiperda*, *S. exigua* and *S. littoralis* caterpillars. The resulting dataset provides extensive insights into the generality and specificity of maize responses to the genus *Spodoptera*. This study reveals the most relevant pathways and metabolites involved in caterpillar‐inducible maize responses and offer a molecular resource for further genetic studies on maize resistance to herbivores.

## 2 Materials and Methods

### 2.1 Plants and Insects

Maize seedlings (*Zea mays* var. Delprim) were grown individually in cylindrical plastic pots (diameter 4 cm; height 10 cm) with commercial potting soil (Profi Substrat soil, Einheitserde, Germany) under greenhouse conditions (27 ± 2°C; 60% relative humidity; supplemental light 16/8 h light/dark photoperiod). All plants used in the experiments were 10–12 days‐old and carried three leaves. Three *Spodoptera* species (Lepidoptera: Noctuidae) were used in the experiments. An in‐house colony of *Spodoptera frugiperda* (JE Smith) was started with insects collected from maize fields in Senegal in 2019, *Spodoptera exigua* (Hübner) eggs were obtained from Entocare (Wageningen, The Netherlands) and *Spodoptera littoralis* (Boisduval) eggs were provided by Syngenta Crop Protection (Stein, Switzerland). All insect colonies were kept under quarantine conditions (FOEN permit A140502) at 24 ± 2°C; 60% relative humidity; 16/8 h light/dark photoperiod and caterpillars were fed on wheat germ‐based artificial diets as described by Maag et al. (2016) and Arce et al. (2021).

### 2.2 Collection of Caterpillar OS

The OS of the three *Spodoptera* species was collected as described by Turlings et al. (1993). Third‐ to fifth‐instar caterpillars of each species were fed on detached maize leaves (Delprim var.) for 24 h prior to the collection. Briefly, to collect OS, caterpillars were gently squeezed near the head to induce regurgitation. Approximately 5–10 µL of OS were collected per larva and the OS collected from the same species were pooled together. The OS was protected from light and was kept on ice during the collection procedure. The crude OS was stored at −80°C until further use. To remove fragments and microorganisms the OS was first centrifuged and then filtered with syringe filters before being used (diameter 13 mm, pore size 0.22 µm, hydrophilic polytetrafluorethylene (PTFE) membrane, BGB Analytik, Switzerland) (Arce et al. 2021) before being used in experiments.

### 2.3 Plant Treatments

**Figure 1:**
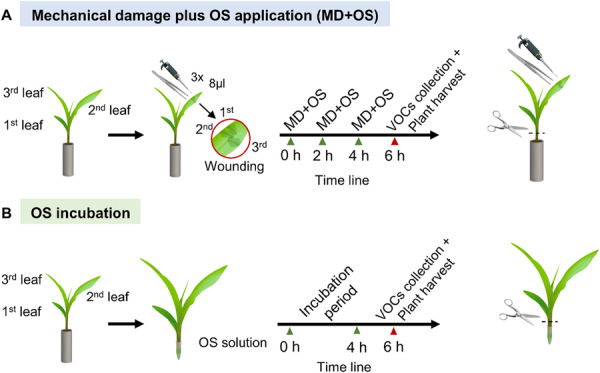
Illustration of the two methods used to treat maize plants with caterpillar oral secretion (OS). Mechanical damage plus oral secretion application experiment (MD + OS): (A) The second and third leaf of three‐leaf stage maize seedlings were wounded three times with serrated forceps and immediately afterwards 8 µL of OS were applied onto the wounded sites. The OS of three *Spodoptera* species (*S. frugiperda*, *S, exigua* and *S. littoralis*) were used. Plants were induced three times over 6 h. Afterwards, the plants were used to sample volatiles and then harvested for phytohormones and transcriptome analyses. OS Incubation experiment: (B) Excised maize leaves were incubated in distilled water with 10% of OS from different *Spodoptera* species for 4 h and then the volatiles were sampled for 2 h. A different batch of similarly treated plants was harvested after 6 h of incubation for phytohormone quantification and transcriptome profiling.

To assess the effect of the OS of the three *Spodoptera* species on maize plant defences, two independent induction experiments were conducted. First, we mimicked herbivory by mechanically damaging the leaves with forceps and then applying OS to the damaged sites (MD + OS). Second, to eliminate the effect of upper‐leaf wounding, we incubated excised seedlings with their leaf‐base (below the first leaf collar) in an OS solution (OS incubation; Figure 1B). The responses measured were phytohormones levels, volatile emissions and the expression of genes known to be involved in maize plant defences.

1. **Mechanical damage experiment**
  Mechanically damaged plants were treated with OS (MD + OS) from the three different caterpillar species to simulate caterpillar feeding (Turlings et al. 1998). Approximately 2 cm^2^ of the second and third true leaf of maize plants were damaged with serrated forceps about 2 cm below the leaf tip on one side of the central vein and 8 µL of OS were immediately applied onto the wounded site (Figure 1A). Maize plants were wounded three times over a 4‐h period, with each wound positioned 1 cm basal to the previous wound site (Figure 1A). The respective treatments with the OS of *S. frugiperda*, *S. exigua*, and *S. littoralis* were labelled as treatments MD‐Sf, MD‐Se, and MD‐Sl, respectively. Control plants were damaged without application of OS (mechanical damage only, MD). After 4 h of treatment, one batch of plants was used to sample the volatiles for 2 h (4–6 h after induction) (Figure 1A). Another different batch of maize plants with the same treatments was harvested 2 h after the last treatment for phytohormone and transcriptomic analyses. For this, the leaves were harvested (excised 1 cm below the first leaf collar, Figure 1A) and immediately flash frozen in liquid nitrogen and stored at −80°C until further analysis.
2. **Incubation experiment**
  For the incubation experiment, as described by Turlings et al. (1993), maize plants were excised 1 cm below the coleoptile (the first visible leaf), and were individually placed in a 1.5 mL microtube that contained either 500 µL of distilled water only or 50 µL of OS from one of the caterpillar species diluted in 450 µL distilled water (Figure 1B). The microtube was covered with aluminum foil and sealed with Parafilm to prevent the degradation and evaporation of the solution. Incubation with distilled water or with diluted OS of *S. frugiperda*, *S. exigua*, and *S. littoralis* are respectively labelled as I‐W, I‐Sf, I‐Se, and I‐Sl. After 4 h of incubation the volatiles were collected for 2 h from each plant individually, and after 6 h of incubation, the basal part of the leaves (approximately 1‐cm long) was removed and the remaining leaf tissue was harvested, flash frozen in liquid nitrogen and stored at −80°C until further analysis (Figure 1B).

### 2.4 Collection and Analysis of Volatiles

To collect volatiles, a multi‐port glass bottle system was used (Arce et al. 2021; Turlings et al. 2000). Plants were individually placed in glass bottles, and the volatiles were trapped on filters containing 25 mg of 80/100 mesh Hayesep‐Q adsorbent (Ohio Valley Specialist Company, Marietta, USA) for 2 h. Charcoal‐filtered air was pushed into the bottom port of the bottle via a Teflon tube (700 L/min) and, after passing over the plant, air was pulled out (400 L/min) via an upper port to which the Hayesep‐Q filter was attached. After each collection, the filters were eluted with 150 µL of dichloromethane (Honeywell, Riedel‐de Haën, DE), and 10 µL of internal standard were added (*n‐*octane and *n*‐nonyl acetate, 20 ng/µL each). Samples were stored at −80°C until further analyses. Six plants per treatment were sampled, for both MD + OS induction and OS incubation.

A gas chromatograph (Agilent 7890B) coupled to a mass‐selective detector (Agilent 5977B GC/MSD) was used to analyze the volatile samples. Two microliters of a sample were injected in pulsed splitless mode onto an Agilent HP‐5MS column (30 m length × 0.25 mm diameter and 0.25 µm film thickness). The oven temperature was started at 40°C and held for 3 min, increased to 100°C at a rate of 8°C min^−1^ and subsequently raised to 200°C, at 5°C min^−1^, followed by a post run period of 3 min at 250°C. Helium was used as a carrier gas and kept at constant flow of 1.1 mL min^−1^. For compound identification we compared the mass spectra to those of commercial standards and NIST 17 library spectra and for quantification we used calibration curves (Arce et al. 2021; Clancy et al. 2023).

### 2.5 Extraction and Quantification of Phytohormones

To evaluate the effect of OS from different *Spodoptera* species on relevant phytohormone levels in maize plants, we quantified JA, jasmonic acid isoleucine (JA‐Ile), SA and ABA as described in Glauser et al. (2011). For this, pooled maize leaf tissues of the MD + OS (*n* = 3; three biological replicates with three plants for each replicate) and OS incubation treatments (*n* = 3; three biological replicates with three plants for each replicate) were ground into a fine powder in liquid nitrogen, of which 100 mg were extracted with 990 µL of ethyl acetate and formic acid (99.5:0.5, v/v). Isotopically labelled hormones were added as internal standards (d5‐JA, d6‐ABA, d6‐SA, and 13C6‐JA‐Ile, 1 ng in 10 µL). Samples were extracted in a mixer mill for 3 min at 30 Hz with 5–8 glass beads (1.25–1.65 mm diameter) and supernatants were collected after centrifugation at 14 000*g* for 3 min. Pellets were reextracted with 500 µL of solvent. Samples were evaporated to dryness and re‐suspended in 200 µL of methanol and water (70:30, v/v).

Phytohormones were analyzed by ultra‐high‐performance liquid chromatography coupled with a tandem mass spectrometry (UHPLC‐MS‐MS) using a protocol slightly adapted from Glauser et al. (2011). The only differences were the use of an Acquity UPLC (Waters) connected to a QTRAP 6500+ (Sciex) and the injection of 2 µL instead of 5 µL used in the original method, which was injected onto an Acquity UPLC BEH C18 column (50 × 2.1 mm, Waters). Quantification was based on internal standardisation using labelled internal standards at a concentration of 5 ng/mL both in the final extracts and in the calibration points.

### 2.6 Total RNA Isolation

Frozen leaf tissues were ground into a fine powder in liquid nitrogen. Tissues from three individual maize seedlings that had received the same treatment were combined into one biological replicate, and three replicates were prepared for each treatment. One hundred milligrams of the pooled leaf powder were then used to extract the total RNA using the GeneJET Plant Purification Mini Kit (Thermo Fisher Scientific Baltics UAB, Vilnius, Lithuania) according to the manufacturer's instructions, and complete DNA removal was performed using the RNase‐Free DNase Set (QIAGEN, Hilden, Germany). RNA degradation and contamination were preliminarily monitored on 1% agarose gel electrophoresis. Further verification of RNA purity was performed using NanoPhotometer® spectrophotometer (IMPLEN, CA, USA). The integrity and quantitation of each RNA sample were assessed by using the RNA Nano 6000 Assay Kit of Agilent 2100 bioanalyzer (Agilent Technologies, Palo Alto, CA, USA).

### 2.7 Library Preparation and Transcriptome Sequencing

A total amount of 1 µg RNA per sample was used for library construction. Sequencing libraries were generated using the NEBNext® Ultra™ RNA Library Prep Kit for Illumina® (NEB, USA) following the manufacturer's recommendations and index codes were added to assign sequences to each sample. The PCR products were purified using the AMPure XP system (Beckman Coulter, Beverly, USA) and library quality was assessed on an Agilent 2100 (Agilent Technologies, Palo Alto, CA, USA). Clustering of the indexed samples was performed on a cBot Cluster Generation System using PE Cluster Kit cBot‐HS (Illumina) according to the manufacturer's instructions. After clustering, the library preparations were sequenced on an Illumina HiSeq. 4000 platform and paired‐end reads (2 × 150 bp) were generated. The raw transcriptome data were deposited in the NCBI short read archive (SRA) under accession number PRJNA889600.

### 2.8 RNA‐Seq Data Analysis

Paired‐end clean reads were mapped to the maize reference genome (B73 RefGen_v4) (Jiao et al. 2017) using HISAT2 v2.0.5 programme (Kim et al. 2015). The expression levels of genes were analyzed using the FeatureCounts programme (Subread v1.5.0 p3) (Liao et al. 2014) with default parameters and they were calculated as fragments per kilobase of transcript per million fragments mapped (FPKM). Differentially expressed genes (DEGs) between different experimental treatments were filtered by using DESeq2 R package v1.20.0 (Love et al. 2014) with false discovery rate (FDR) < 0.05 and an absolute value of log2‐transformed fold change (treatment/control) > 1. KEGG (Kyoto Encyclopedia of Genes and Genomes) pathway enrichment was analyzed using ClusterProfiler v3.8.1 (Yu et al. 2012) (pathways with adjusted *p* < 0.05 were considered significantly enriched).

### 2.9 Statistical Analyses

Statistical analyses were performed in R (v. 4.0.0; R Foundation for Statistical Computing, Vienna, Austria). Linear Models (LM) with a Gaussian distribution were used to verify the differences among treatments in phytohormone and volatile profiles. Estimated Marginal means (EMMeans) were used to compare significant differences between specific treatments.

## 3 Results

### 3.1 General Transcriptomic Response

#### 3.1.1 Overview of Transcriptional Changes in Maize Plants in Response to OS of Different *Spodoptera* Species Under Different Types of Induction

**Figure 2:**
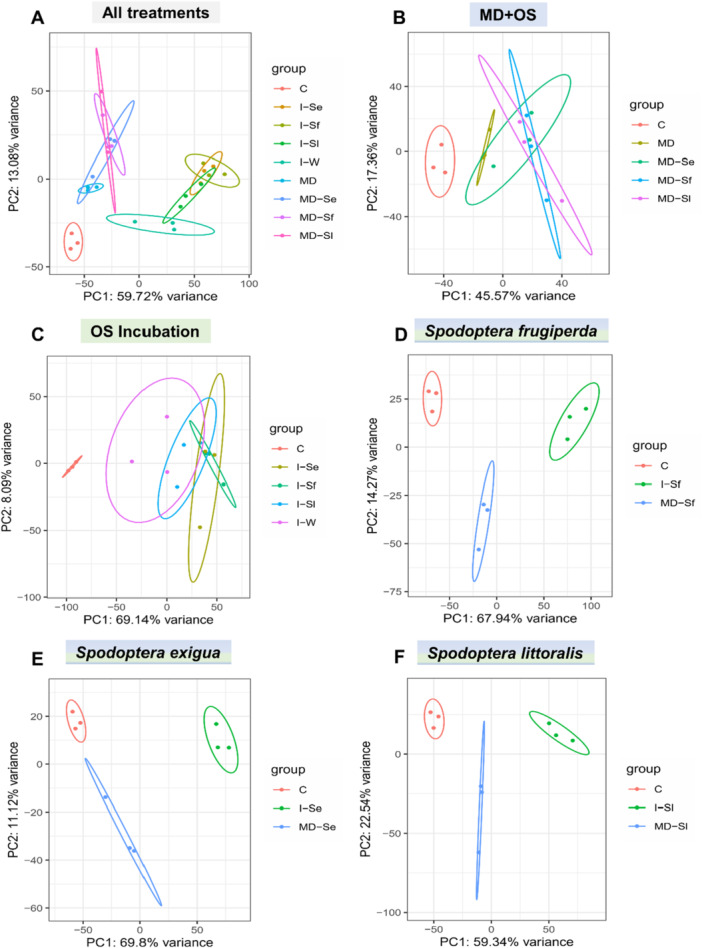
Principal component analyses (PCA) of maize transcriptomes after induction with oral secretion (OS) of different *Spodoptera* species. Plants were subjected to the following treatments in the mechanical damage plus OS application experiment (MD + OS): mechanically damaged (MD), mechanically damaged and application of OS of *Spodoptera frugiperda* (MD‐Sf), *Spodoptera exigua* (MD‐Se), or *Spodoptera littoralis* (MD‐Sl). Treatments in the OS incubation experiment: incubation of cut maize leaves in distilled water (I‐W) or in 10% OS of *S. frugiperda* (I‐Sf), *S. exigua* (I‐Se), or *S. littoralis* (I‐Sl). Healthy, untreated seedlings served as controls (C). (A) PCA plot of transcripts of maize in response to the different treatments. (B and C) PCA plots of maize transcriptomes in response to different treatments within the MD + OS experiment (B) and the OS incubation experiment (C). (D–F) PCA plots of transcripts of maize in response to OS of *S. frugiperda* (D), *S. exigua* (E) and *S. littoralis* (F) across experiments. Ellipses show 95% confidence intervals. For detailed information on top and bottom loadings (genes associated with positive or negative values on principal component) for each PCA plot, refer to Supporting Information S5: Table S3.

A total of 34 567 transcripts from 46 430 predicted genes in the B73 V4 reference genome were detected across all 27 samples (Supporting Information S1: Data S1). Detailed information on RNA sequencing and mapping is provided in Supporting Information S3: Table S1. Principal component analysis (PCA) revealed distinct patterns in the maize leaf transcriptome following different induction treatments (Figure 2A and Supporting Information S4: Table S2). Genes involved in water deprivation response (dehydrin), defence response (benzoxazinone synthesis 10 and 14), transcription regulation (rRNA N‐glycosidase, homeobox‐transcription factor 41) and fundamental metabolism process (asparagine synthetase 3) explained most of the variance in the first principal component (PC1). Genes involved in biosynthesis of terpenoid and isoprenoid (TPS 2, 3, 10 and 1‐deoxy‐d‐xylulose‐5‐phosphate synthase), oxidative stress response (peroxidase), amino acid and sugar transport, and cytochrome P450 family genes explained most of the variance in the second principal component (PC2) (Supporting Information S5: Table S3). For each *Spodoptera* species, the transcriptomic profile varied depending on the method of induction (Figure 2D–F). However, within each induction method, there were no clear differences among the transcriptomic profiles of maize plants treated with OS of different *Spodoptera* species (Figure 2B,C). TPS 10, crucial for the induced emissions of various sesquiterpenes in maize, always contributed most to the separation in PC1 for each induction method. Detailed information on top and bottom loadings (genes associated with positive or negative values on principal component) for each PCA plot is provided in Supporting Information S5: Table S3.

**Figure 3:**
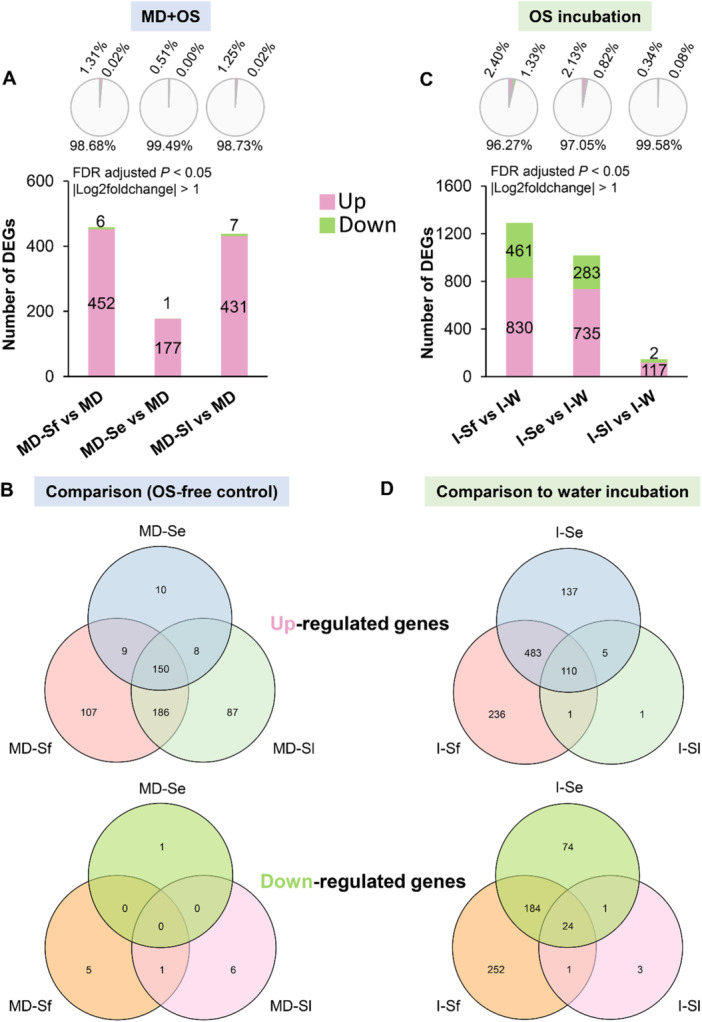
Differentially expressed genes (DEGs) in maize leaves in response to treatment with oral secretion (OS) of different *Spodoptera* species compared with OS‐free controls. (A) Total number of DEGs that were significantly upregulated or downregulated in maize leaves in response to mechanical leaf damage plus application of OS from *S. frugiperda* (MD‐Sf), *S. exigua* (MD‐Se), or *S. littoralis* (MD‐Sl) compared with mechanical damage alone (MD). The pie chart indicates the percentage of DEGs respectively regulated by MD‐Sf, MD‐Se, or MD‐Sl for all 34 567 present genes across 27 cDNA libraries. (B) Venn diagram illustrating the number of specifically and commonly upregulated and downregulated DEGs in maize in response to mechanical damage plus application of OS from different *Spodoptera* species. (C) Total number of DEGs that were significantly upregulated or downregulated in maize leaves after incubation in the OS of *S. frugiperda* (I‐Sf), *S. exigua* (I‐Se), or *S. littoralis* (I‐Sl) compared with mere water incubation (I‐W). The pie chart indicates the percentage of DEGs, respectively, regulated by I‐Sf, I‐Se, and I‐Sl for all 34 567 present genes across 27 cDNA libraries. (D) Venn diagram illustrates the number of specifically and commonly regulated DEGs in maize in response to incubation in OS of different *Spodoptera* species.

The number of DEGs between each treatment and the specific OS‐free control was calculated in both mechanical damage plus OS application (MD + OS) and OS incubation experiments (Supporting Information S1: Data S2). In the mechanical damage plus OS application experiment, we analyzed the DEGs of OS‐treated plants relative to plants with mechanical damage only. Compared with wounding alone, applying OS of *S. frugiperda*, *S. exigua* and *S. littoralis* to mechanical wounds induced 458, 178 and 438 DEGs, respectively (Figure 3A). A total of 150 genes were upregulated by applying OS of three different *Spodoptera* species to wounded leaves. Moreover, OS of *S. frugiperda* and *S. littoralis* specifically resulted in more DEGs than *S. exigua* OS (Figure 3B and Supporting Information S1: Data S3). The expression of only 5, 1 and 6 genes was lower in wounded leaves respectively treated with OS of *S. frugiperda*, *S. exigua* and *S. littoralis* compared with mechanical damage alone (Figure 3B and Supporting Information S1: Data S3). In the incubation experiment, we compared the DEGs of OS‐incubated plants relative to water‐incubated plants. Incubation in OS of *S. frugiperda*, *S. exigua* and *S. littoralis* resulted in 1291, 1018 and 146 DEGs, respectively, compared to water incubation (Figure 3C). *Spodoptera frugiperda* OS incubation specifically affected the expression of more genes (236 up and 252 down) than incubation in OS of *S. exigua* (137 up and 74 down) and *S. littoralis* (1 up and 3 down) (Figure 3D and Supporting Information S1: Data S3). Supporting Information S2 and S1: Figure S1 and Data S4 provide detailed information on the DEGs between each treatment (type of induction and caterpillar species) and the unmanipulated control.

#### 3.1.2 Differentially Expression of Genes in Plants Induced by OS From Different *Spodoptera* Species

**Figure 4:**
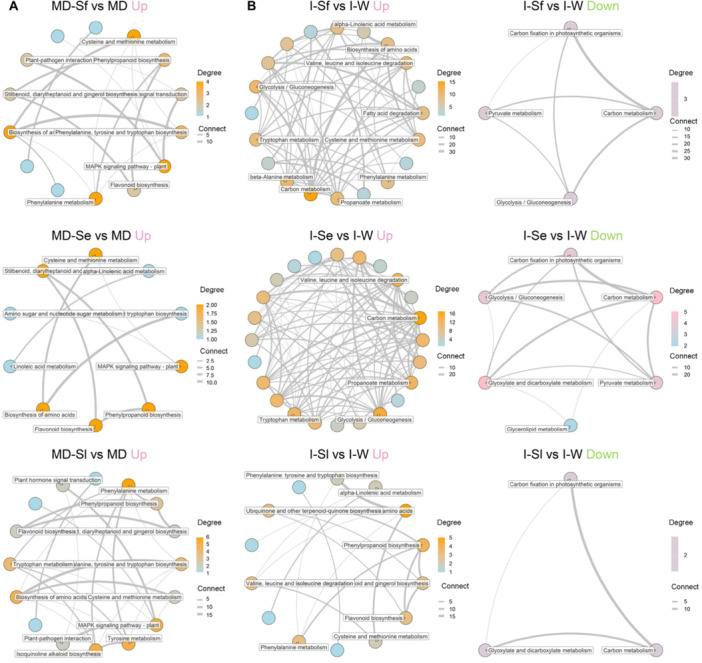
KEGG (Kyoto Encyclopedia of Genes and Genomes) network of the key pathways of differentially expressed genes (DEGs) in maize induced by different treatments compared with corresponding control in mechanical damage plus oral secretion (OS) application and OS incubation experiment. (A) Network of the upregulated KEGG pathways in maize, comparing mechanical damage plus application of OS of *S. frugiperda* (MD‐Sf), *S. exigua* (MD‐Se), *S. littoralis* (MD‐Sl), and mechanical damage alone (MD). (B) Network of the upregulated and downregulated KEGG pathways in maize, comparing incubation in OS of *S. frugiperda* (I‐Sf), *S. exigua* (I‐Se), *S. littoralis* (I‐Sl), and water (I‐W). The grey lines indicate connections between pathways. Colour coding represents the degree of nodes. For full datasets, refer to Supporting Information S6: Table S4.

The DEGs between each treatment and the specific OS‐free control were further subjected to KEGG pathway enrichment analysis and network analysis to identify pathways that are differentially regulated (Figure 4 and Supporting Information S2: Figure S2). Compared with mechanical wounding alone, applying OS of *S. frugiperda*, *S. exigua*, and *S. littoralis* to wounded leaf regulated 10, 8 and 11 KEGG pathways, respectively, and the pathways induced by different caterpillar OS overlapped considerably (Supporting Information S2 and S6: Figure S2A–C and Table S4). The biosynthesis of flavonoids, monoterpenoids, and phenylpropanoids and the metabolism of α‐linolenic acid, as well as other metabolic pathways associated with primary metabolism, showed significant changes in maize leaves after caterpillar OS application (Supporting Information S2 and S6: Figure S2A–C and Table S4). Biosynthesis of amino acids, phenylalanine metabolism, and MAPK signalling pathway were the central upregulated pathways in maize leaves after wounding plus OS treatment. The cysteine and methionine metabolism pathway was one of the central pathways upregulated after application of OS of *S. frugiperda* and *S. exigua*, whereas tyrosine metabolism and isoquinoline alkaloid biosynthesis pathways occupied a more central position in the network of upregulated pathways in wounded maize leaves treated with OS of *S. littoralis* compared to the other two *Spodoptera* species (Figure 4A).

When comparing OS incubation and water incubation treatments, the DEGs of plants incubated in OS of *S. frugiperda*, *S. exigua*, and *S. littoralis* were assigned to 21, 18 and 9 pathways, respectively, mainly involving α‐linolenic acid metabolism and the biosynthesis of phenylpropanoids and flavonoids, and some primary metabolic pathways, including the metabolism of amino acids and carbohydrates. Several DEGs of plants incubated in OS of *S. frugiperda* and *S. exigua* are also involved in the transduction of plant hormone signals (Supporting Information S2 and S6: Figure S2D–F and Table S4). Pathways involved in carbon metabolism, glycolysis/gluconeogenesis, valine, leucine and isoleucine degradation were the core up‐regulated pathways in maize incubated in OS of *S. frugiperda* and *S. exigua*, whereas biosynthesis of amino acids and phenylpropanoid biosynthesis were the mostly up‐regulated in maize incubated in OS of *S. littoralis*. Fundamental metabolism pathways such as the carbon metabolism pathway were always the core down‐regulated pathways after OS incubation treatment (Figure 4B).

### 3.2 Hormonal Response

#### 3.2.1 Phytohormone‐Related Genes Induced by OS of Different *Spodoptera* Species

**Figure 5:**
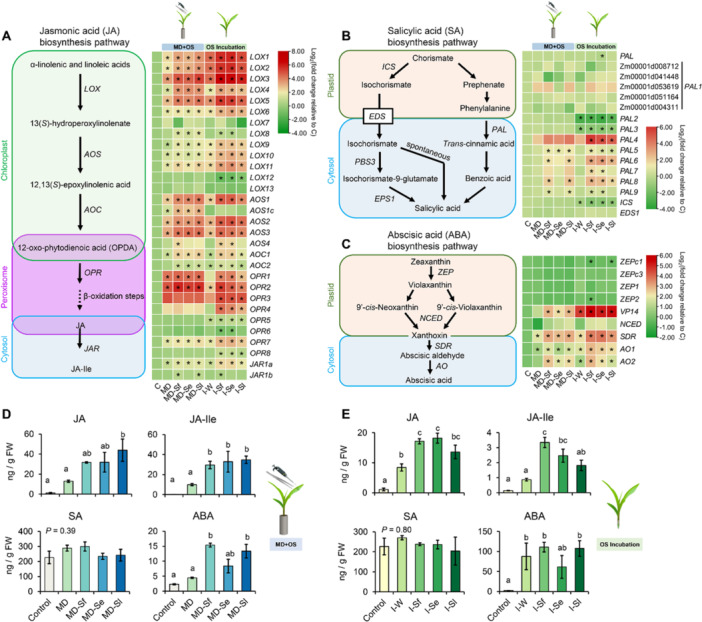
Effects of treatment with oral secretion (OS) of different *Spodoptera* species on plant hormone‐related gene expression and phytohormones in maize. Plants were subjected to the following treatments in the mechanical damage plus OS application experiment (MD + OS): mechanically damaged (MD), mechanically damaged and application of OS of *Spodoptera frugiperda* (MD‐Sf), *Spodoptera exigua* (MD‐Se), or *Spodoptera littoralis* (MD‐Sl). Treatments in the OS incubation experiment: incubation of cut maize leaves in distilled water (I‐W) or in 10% OS of *S. frugiperda* (I‐Sf), *S. exigua* (I‐Se), or *S. littoralis* (I‐Sl). Control plants (C) were kept untreated. Schematic diagram and heat map depict the biosynthesis pathway and the gene expression pattern of jasmonic acid (A), salicylic acid (B), and abscisic acid (C) pathways. Colour coding represents the range of log_2_(fold change relative to control). Genes differentially expressed between control and treatments are indicated by stars (false discovery rate [FDR] adjusted *p* < 0.05). For full datasets, refer to Supporting Information S7: Table S5. Concentrations of jasmonic acid (JA), jasmonoyl‐isoleucine (JA‐Ile), salicylic acid (SA) and abscisic acid (ABA) in maize leaves after treatments of MD + OS experiment (D) or OS incubation experiment (E) are shown in nanograms/FW: fresh weight. Error bars represent standard error of the mean (for H‐C, *n* = 2; for all the other treatments, *n* = 3). Different letters indicate significant differences among treatments (*p* < 0.05). *p* Values are given for treatment comparisons [GLM (family, Gaussian)], followed by pairwise comparisons of EMMeans.

Many genes involved in phytohormone biosynthesis were induced in response to all OS treatments. Incubation treatments induced plant phytohormone‐related genes to a greater extent than OS application to wounded leaves. However, within each induction method, there was no clear difference among the expression pattern of plant phytohormone‐related genes in maize treated with OS from different *Spodoptera* species (Figure 5 and Supporting Information S7: Table S5).

13‐Lipoxygenases (LOXs) such as ZmLOX8 and ZmLOX10, initiate the biosynthesis of JA and related metabolites (Christensen et al. 2013). In addition, 9‐LOX enzymes, such as ZmLOX5, also play a signalling role in maize defence against herbivory by modulating the production of wound‐induced oxylipins and insecticidal compounds (Yuan et al. 2023). Our results showed that both induction methods strongly increased the expression of 9‐lipoxygenase genes (especially *LOX1*, *LOX2*, *LOX3*, *LOX4* and *LOX5*). Most of the transcripts of allene oxide synthase (*AOS*), allene oxide cyclase (*AOC*) and oxo‐phytodienoate reductase (*OPR*) were also increased after OS treatments. OS incubation induced the expression of three 13‐lipoxygenase genes (*LOX9*, *LOX10* and *LOX11*) to a greater extent than OS application to wounded leaves (Figure 5A).

SA is biosynthesized through two distinct pathways: the isochorismate synthase (ICS) pathway and the phenylalanine ammonia‐lyase (PAL) pathway. PAL catalyzes the conversion of l‐phenylalanine to ammonia and trans‐cinnamic acid, playing an essential role in the biosynthesis of phenolic compounds (Zheng et al. 2024). We found that the expression of several *PAL* genes (*PAL5*, *PAL6*, *PAL8* and *PAL9*) was clearly upregulated in maize leaves after application of OS of *S. frugiperda* and *S. littoralis*, whereas only one *PAL* gene (*PAL5*) was slightly induced after applying *S. exigua* OS to wounded leaves. The transcription of *PAL4* was slightly induced after OS application but was not different from the control. OS incubation induced four *PAL* genes (*PAL4*, *PAL5*, *PAL6* and *PAL8*) but significantly suppressed the expression of *PAL2*, *PAL3* and *ICS* (Figure 5B).

OS Application to wounded leaves and OS incubation strongly increased the expression of the key regulator *VP14* and several other genes (*SDR*, *AO1* and *AO2*) that are involved in ABA biosynthesis. Mechanical leaf damage alone only slightly induced the expression of *SDR* and *AO1* (Figure 5C). Three genes involved in ethylene biosynthesis (*ZmACS2*, *ZmACO15* and *ZmACO31*) were also upregulated upon application of the different OS (Supporting Information S2: Figure S3) but not by mechanical damaged by itself. In fact, just damage did not induce any gene involved in the ethylene pathway. OS incubation strongly increased the transcripts of three ACS genes (*ZmACS2*, *ZmACS6* and *ZmACS31*) while the expression of the ethylene receptor *ZmETR2* and the transcription factor *EIN2* were respectively up‐ and downregulated in response to OS incubation (Supporting Information S2: Figure S3).

#### 3.2.2 Phytohormones Induced by OS of Different *Spodoptera* Species

To determine phytohormone changes in response to the OS of different *Spodoptera* species, we analyzed the concentrations of JA, JA‐isoleucine (JA‐Ile), SA, and ABA in maize leaves following two independent induction experiments (Figure 5D,E). Application of OS from different *Spodoptera* species to wounded sites induced similar levels of JA, JA‐Ile and ABA. More specifically, compared to mechanical wounding alone, applying *S. littoralis* OS significantly increased the level of JA. OS of all *Spodoptera* species induced higher levels of JA‐Ile, whereas application of OS from *S. frugiperda* and *S. littoralis* significantly increased the concentration of ABA in maize leaves (Figure 5D). Incubation in OS from different *Spodoptera* species caused similar increases in JA concentrations. Compared with water incubation, incubation in OS from *S. frugiperda* or *S. exigua* induced higher levels of JA and JA‐Ile, whereas OS from *S. littoralis* did not affect these levels. In addition, the level of JA‐Ile in maize leaves incubated in *S. frugiperda* OS was significantly higher than in plants incubated in *S. littoralis* OS. ABA production was dramatically increased by both water incubation and OS incubation, and different OS induced similar levels of ABA (Figure 5E). Neither OS application nor OS incubation affected SA concentrations in maize leaves (Figure 5D,E).

### 3.3 Non‐Volatile Defence Metabolites

#### 3.3.1 BXs Biosynthesis‐Related Genes Induced by OS From Different *Spodoptera* Species

**Figure 6:**
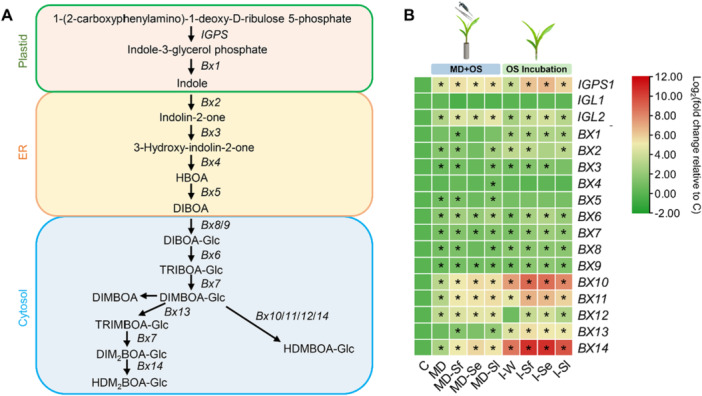
Effects of treatment with oral secretion (OS) of different *Spodoptera* species on benzoxazinoid (BX) biosynthesis pathway gene expression. Plants were subjected to the following treatments in the mechanical damage plus OS application experiment (MD + OS): mechanically damaged (MD), mechanically damaged and application of OS of *Spodoptera frugiperda* (MD‐Sf), *Spodoptera exigua* (MD‐Se), or *Spodoptera littoralis* (MD‐Sl). Treatments in the OS incubation experiment: incubation of cut maize leaves in distilled water (I‐W) or in 10% OS of *S. frugiperda* (I‐Sf), *S. exigua* (I‐Se), or *S. littoralis* (I‐Sl). Control plants (C) were kept untreated. (A) Schematic diagram of the BX biosynthesis pathway (modified from Tzin et al. 2017). ER, endoplasmic reticulum. (B) Fold changes of genes involved in BX biosynthesis relative to control. Genes differentially expressed between control and treatments are indicated by stars (false discovery rate [FDR] adjusted *p* < 0.05). For full datasets, refer to Supporting Information S8: Table S6.

We further assessed the expression of genes involved in BX biosynthesis (Figure 6 and Supporting Information S8: Table S6). Compared with undamaged control, mechanical damage plus OS application in general strongly induced most of the genes involved in BX biosynthesis in maize leaves. This was particularly evident for *IGPS* and a *BX1* homolog (*BX1‐igl2*), which are involved in indole production, as well as several *BX* genes responsible for the synthesis of HDMBOA‐Glc (*BX10*, *BX11*, *BX12* and *BX14*) and HDM_2_BOA‐Glc (*BX14*) (Figure 6B). OS incubation induced the expression of several *BX* genes (*IGPS*, *BX10*, *BX11*, *BX13* and *BX14*) to a greater extent than mechanical damage plus OS application (Figure 6B).

We also compared the response to the different OS for each *BX* gene in maize leaves. In the mechanical damage plus OS application experiment, all OS usually induced similar expression of *BX* genes compared to wounding alone. Only a few *BX* genes showed some differences when treated with OS from different *Spodoptera* species. For instance, *S. exigua* OS significantly induced the expression of *BX10* and *BX14*, whereas *S. littoralis* OS significantly induced *BX13* compared to plants that were only wounded (Supporting Information S2: Figure S4A). The expression of *BX10* and *BX14* were also induced by OS of *S. frugiperda* and *S. littoralis*, but this did not differ from mechanical damage alone (Supporting Information S2: Figure S4A). In the incubation experiment, OS of *S. frugiperda* or *S. exigua* increased the expression of *IGPS*, *BX9* and *BX11* compared to the water control (Supporting Information S2: Figure S4B). Incubation in OS of *S. frugiperda* or *S. exigua* also induced *BX10* and *BX14* but this did not differ from water incubation. *S. exigua* OS induced the expression of *BX7* more strongly than *S. frugiperda* and *S. littoralis* OS, both of which did not show any difference from water incubation (Supporting Information S2: Figure S4B).

### 3.4 Volatile Metabolites

#### 3.4.1 Volatile Terpene Biosynthesis‐Related Genes Induced by OS of Different *Spodoptera* Species

**Figure 7:**
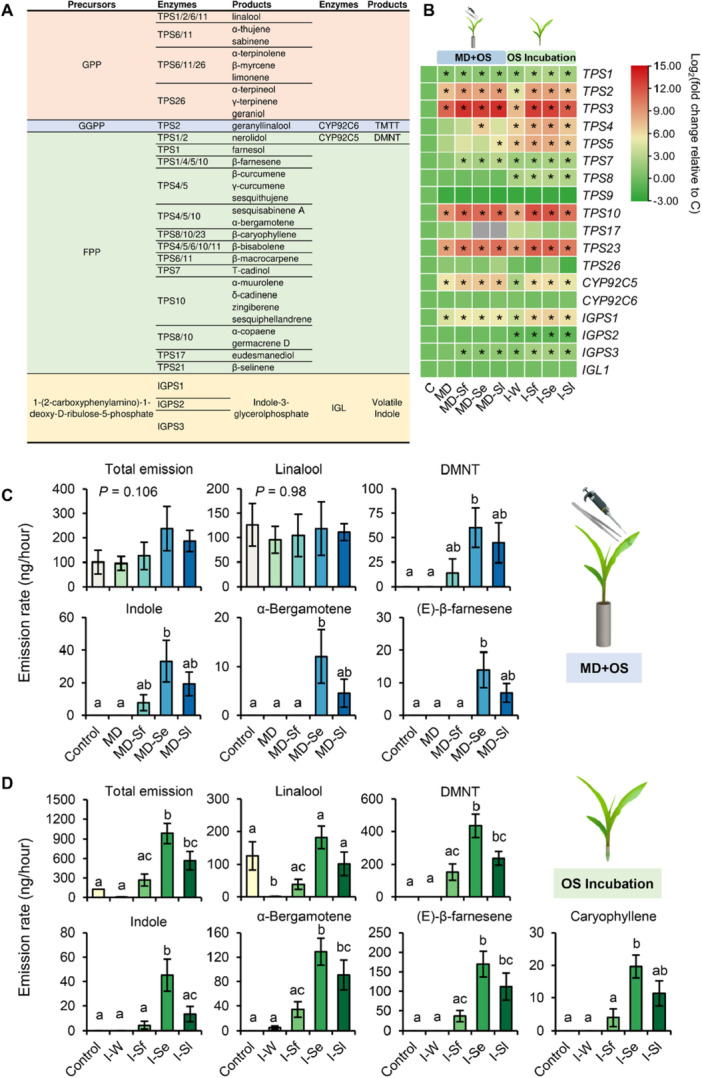
Effects of treatment with oral secretion (OS) of different *Spodoptera* species on volatile biosynthesis gene expression and maize volatile emissions. Plants were subjected to the following treatments in the mechanical damage plus OS application experiment (MD + OS): mechanically damaged (MD), mechanically damaged and application of OS of *Spodoptera frugiperda* (MD‐Sf), *Spodoptera exigua* (MD‐Se), or *Spodoptera littoralis* (MD‐Sl). Treatments in the OS incubation experiment: incubation of cut maize leaves in distilled water (I‐W) or in 10% OS of *S. frugiperda* (I‐Sf), *S. exigua* (I‐Se), or *S. littoralis* (I‐Sl). Control plants were kept non‐manipulated. (A) Volatile terpene and indole biosynthesis‐related enzymes in maize (Block et al. 2019; Richter et al. 2021). Precursors: GPP, geranyl diphosphate; GGPP, geranylgeranyl diphosphate; FPP, farnesyl diphosphate. Enzymes: TPS, terpene synthase; CYP92C5 and CYP92C6, cytochrome P450 monooxygenases; IGPS, Indole‐3‐glycerol phosphate synthase; IGL, tryptophan synthase A homolog1. Two irregular acyclic homoterpene volatile products: DMNT, (E)‐3,8‐dimethyl‐1,4,7‐nonatriene; TMTT, (E,E)‐4,8,12‐trimethyltrideca‐1,3,7,11‐tetraene. (B) Fold changes of genes involved in volatile terpene and indole biosynthesis relative to control. Genes differentially expressed between control and treatments are indicated by stars (false discovery rate (FDR) adjusted *p* < 0.05). Grey block, no value available. For full datasets, refer to Supporting Information S8: Table S6. (C and D) Average of the total volatile emission and the most representative compounds emitted by maize plants after treatments of MD + OS (C) or OS incubation (D). Error bars represent standard error of the mean (*n* = 6). Different letters indicate significant differences among treatments (ANOVA, pairwise comparisons of EMMeans, *p* < 0.05).

We also analyzed the specific expression patterns of volatile terpene biosynthesis‐related genes in maize leaves in response to OS from different *Spodoptera* species (Figure 7 and Supporting Information S8: Table S6). Compared with the undamaged control, mechanical damage plus OS application and OS incubation significantly upregulated the expression of five TPS genes (*TPS1*, *TPS7*, and especially *TPS2*, *TPS10* and *TPS23*) and one cytochrome P450 monooxygenase gene (*CYP92C5*) that is responsible for DMNT production (Figure 7B). However, all these genes except *TPS7* were also highly induced upon mechanical leaf damage alone (Figure 7B). OS incubation also upregulated the expression of *TPS4*, *TPS5* and *TPS8* in maize leaves (Figure 7B).

The OS from different *Spodoptera* species induced a similar expression pattern of *TPS* genes (Supporting Information S2: Figure S5A). When applied to damaged sites each OS induced the expression of *TPS2* and *TPS3*, but for *S. exigua* OS this induction was less pronounced and not different from mechanical damage alone. Treatment with different OS also induced the expression of *TPS23*, but the induction by OS from *S. frugiperda* and *S. exigua* was not different from wounding alone. The *TPS10* gene was highly induced by OS application on wounded leaves but did not different from the wounding alone treatment (Supporting Information S2: Figure S5A). The only gene that was differentially upregulated among the treatments with OS from the different *Spodoptera* species was *TPS4*, which was much more strongly induced after *S. exigua* OS application compared to OS from *S. frugiperda* and *S. littoralis* (Supporting Information S2: Figure S5A). In the incubation experiment, the OS of *S. frugiperda* and *S. exigua* slightly upregulated the transcription of various *TPS* genes (e.g., *TPS1, TPS2*, *TPS4*, *TPS5, TPS10* and *TPS23*) in maize leaves compared to water incubation and OS from *S. littoralis* (Supporting Information S2: Figure S5B).

#### 3.4.2 The OS of Different *Spodoptera* Species Induce Quantitatively Different Volatile Emissions

Lastly, we measured the volatiles emitted by maize plants treated with OS from different *Spodoptera* species. In response to mechanical damage plus OS application from either of the three different *Spodoptera* species, all plants started to release indole and the homoterpene DMNT, whereas the sesquiterpenes α‐bergamotene and (*E*)‐β‐farnesene were only emitted by plants treated with OS from *S. exigua* and *S. littoralis* (Figure 7C). Mechanical leaf damage alone or wounding plus OS application did not affect the emission of linalool (Figure 7C), which was already released in significant amounts by untreated maize plants. In the incubation experiment, all OS‐incubated plants initiated the release of DMNT, indole, caryophyllene, α‐bergamotene and (*E*)‐β‐farnesene. The emission of linalool was strongly reduced upon water incubation but was restored when plants were incubated in OS, independent of the source of the OS (Figure 7D). Interestingly, plants incubated in *S. exigua* OS always emitted higher amounts of volatiles than those incubated in *S. frugiperda* OS (Figure 7D). In both types of OS application and OS incubation experiments there was a similar trend that plants treated with *S. exigua* OS showed a more pronounced volatile emissions than those treated with OS from the other two *Spodoptera* species. Of the three, the induction with *S. frugiperda* OS consistently resulted in the least pronounced increases in volatile emissions (Figure 7C,D).

## 4 Discussion

By profiling changes in transcriptome, phytohormones, and volatile emissions in maize, we studied the specifics of defence responses in maize plants to three closely related *Spodoptera* species, *S. frugiperda*, *S. exigua* and *S. littoralis*, with two independent induction methods (mechanical damage plus OS application and OS incubation). The results reveal that caterpillar OS from different *Spodoptera* species trigger overall similar changes in the maize transcriptome and phytohormone levels but differ in causing specific gene expression and how they modulate volatile emissions.

Treatments with OS from *Spodoptera* caterpillars triggered greater changes in the transcriptome, phytohormones, and volatile emissions in maize than OS‐free treatments (artificial damage and water incubation). Elicitation with OS caused more upregulated DEGs than downregulated DEGs in maize leaves (Figure 3A,C). This is similar to transcriptome responses reported for herbivory by *S. frugiperda* (Ye et al. 2022) and *S. exigua* (Tzin et al. 2017), confirming that elicitors in *Spodoptera* caterpillar OS play a critical role in activating defence responses in maize. Thus far, the well‐studied elicitors volicitin and inceptin have been identified in the OS of *Spodoptera* caterpillars (Alborn et al. 1997; Schmelz et al. 2006; Turlings et al. 2000; Steinbrenner et al. 2020). Yet, little is known about the composition of *Spodoptera* OS and their associations with plant defence responses, but it has been shown that there is variability in the mix of FAC‐type elicitors among distantly related lepidopteran species (Pohnert et al. 1999; Spiteller et al. 2001; Mori et al. 2003). A recent study also has shown that the FACs content and GOX activity in OS differs among different lepidopteran species and that the quantity of OS‐derived FACs determines the intensity of volatile emissions in maize (Ling et al. 2021). In this study, elicitation by OS from *S. frugiperda* specifically induced more genes than OS from *S. exigua* and *S. littoralis* (Figure 3B,D), suggesting that *S. frugiperda* OS may contain larger amounts or more potent elicitors than OS from the other two species. Importantly and surprisingly, this did not result in higher volatile emissions.

The metabolome and microbiome of the three *Spodoptera* species are highly plastic and influenced by factors such as food source and geographical distribution. Their respective OS contain different numbers and types of proteins potentially involved in digestion (e.g., proteases) and proteins may be linked to plant‐insect interactions (e.g., polycalin). Previous work with *S. littoralis* found that, compared to OS from caterpillars fed on artificial diet, OS from pepper and tomato fed caterpillars showed a decreased abundance of peroxidases. In contrast, the amounts of peroxidase increased in OS from *S. frugiperda* and *S. exigua* larvae fed on these plants (García‐Marín et al. 2025; Zhang et al. 2024). Caterpillars may directly alter plant defensive responses through these proteins or indirectly change plant volatile emission by modulating the levels of FACs through the regulation of biosynthesis and hydrolysis enzymes (Ling et al. 2021). The gut microbial composition, in particular of *Enterococcus* and *Pseudomonas*, may also be implicated in plant defence responses. *Enterococcus* are known to be the dominant bacteria OS of *S. frugiperda*, (Leclerc et al. 2024) and have identified in *S. exigua*, whereas in *S. littoralis* larvae, the core microbial community has been found to comprise *Enterococcus*, *Lactobacillus*, and *Clostridium* (Shao et al. 2024). The gut microbiome of Lepidoptera can mitigate plant defences by interfering with the plant's perception of herbivory. For instance, antibiotic‐treated *S. frugiperda* larvae with reduced gut microbial populations trigger stronger plant responses and certain bacteria isolated from their OS (e.g., *Enterobacter*, *Pantoea*, and *Rahnella*) can directly suppress plant defence enzymes like peroxidase and protease inhibitors in tomato (Acevedo et al. 2017). Further metabolomic and functional studies are needed to fully characterise these differences and the role of these microbes in plant‐insect interactions.

Applying OS of *S. frugiperda* and *S. littoralis* to wounded maize leaves induced more DEGs than *S. exigua* OS (Figure 3A). In contrast, maize leaves incubated in OS of *S. frugiperda* and *S. exigua* exhibited more DEGs than those incubated in *S. littoralis* OS (Figure 3C). There could be several explanations for the divergence of transcriptional changes in maize between the two induction methods. For instance, the uptake efficiency of OS‐derived elicitors such as FACs might be different between external application of OS to wounded leaves and OS incubation. Additionally, aside from FACs such as volicitin, OS of *Spodoptera* caterpillars also contain eliciting proteins, including GOX (Consales et al. 2012; Ling et al. 2021) and porin‐like proteins (Guo et al. 2013) that mediate defence response in plants. Because GOX levels dictate the strength and timing of stomatal and volatile responses, the plant's responses are typically herbivore‐specific and not uniform (Lin et al. 2021, 2022; Gao et al. 2024; Seidl‐Adams et al. 2015; Jones et al. 2023; Poretsky et al. 2021). This species‐dependent modulation complicates efforts to pinpoint universal maize defence patterns across caterpillar guilds. These proteins could be unstable and during in vitro treatments they might degrade, leading to different plant defence responses. Finally, it is possible that a transient transcriptomic change in the leaves only occurs early during *S. littoralis* OS incubation and fades out after 6 h of incubation.

The phytohormones JA, SA, ABA and ET are important regulators of plant defences against insect herbivores (Erb et al. 2012; Wu and Baldwin 2010). JA signalling plays a central role in the induction and regulation of defence responses against chewing insects (Lortzing and Steppuhn 2016). ABA and ET are stress‐related hormones and their role in modulating plant defence and resistance is also well documented (Broekgaarden et al. 2015; Olds et al. 2018). In maize plants, feeding by *S. exigua* increases the levels of JA, JA‐Ile, and ABA (Tzin et al. 2017) and stimulates the production of ET (Schmelz et al. 2003). Feeding by *S. littoralis* induces strong increases of JA and a relatively modest induction of ABA in maize leaves (Erb et al. 2009). Application of *Mythimna separata* OS to wounded maize leaf increases the levels of JA, JA‐Ile, ABA and ET in abundance (Qi et al. 2016). Here, the amounts of JA, JA‐Ile and ABA increased in response to elicitation by OS of *Spodoptera* caterpillars (Figure 5). Several critical genes involved in the biosynthesis of JA, ABA and ET were also found to be highly upregulated upon OS elicitation (Figure 5). These results confirm that JA, ABA and ET signalling are involved in the response of maize to *Spodoptera* caterpillars. It should be noted that we did not follow the time‐course of the insect‐induced phytohormone changes, therefore we can only draw conclusions about the differential activity of OS of the three *Spodoptera* species in phytohormone induction at one particular timepoint.

SA is involved in plant immunity against herbivores and pathogens, and the induction of SA levels in plants to insects can be highly herbivore species‐specific and time‐dependent (Leitner et al. 2005; De Vos et al. 2005; Diezel et al. 2009). Application of OS of *M. separata* to maize leaf wound sites for 0.5–6 h elicits SA accumulation (Qi et al. 2016). However, infestation by *Ostrinia furnacalis*, *S. exigua*, or *S. littoralis* do not increase SA concentration in maize leaves (Erb et al. 2009; Tzin et al. 2017; Guo et al. 2019). Here we confirmed that exposure to OS of different *Spodoptera* has no effect on SA concentrations (Figure 5), indicating that *Spodoptera* caterpillars do not activate SA‐dependent signalling in maize. The biosynthesis of SA is independently regulated by the ICS and PAL pathways (Dempsey et al. 2011). Mechanical damage alone had little impact on the expression of SA biosynthetic genes, whereas applying caterpillar OS to wounded leaves strongly induced the expression of several *PAL* genes (Figure 5B). This suggests that elicitors in the OS of *Spodoptera* caterpillars rather than mechanical wounding trigger PAL‐related phenylpropanoid metabolism in maize plants. Hydroxycinnamic acid amides represent a group of specialised phenylpropanoid metabolites that function in plant resistance to abiotic and biotic stress (Zeiss et al. 2021). *Spodoptera littoralis* infestation is known to strongly increase the abundance of several hydroxycinnamic acid amide derivatives such as coumaroyl‐ and feruloyl‐tyramine conjugates, and coumaroyl‐tryptamine (Marti et al. 2013). Interestingly, applying OS of *S. frugiperda* and *S. littoralis* to wounded leaves induced *PAL* genes to a greater extent than *S. exigua* OS application, whereas incubation in OS of *S. frugiperda* and *S. exigua* had a stronger impact on the transcription of both *PAL* and *ICS* genes than incubated in *S. littoralis* OS (Figure 5B). These results suggest the presence of different active compounds in the different *Spodoptera* OS that induce phenylpropanoid metabolism, and the activity might also be different because of differences in within‐plant transport of these compounds.

The role of BXs in defence against herbivorous insect has been extensively studied in maize (Frey et al. 2009; Maag et al. 2016; Tzin et al. 2017). Feeding by lepidopteran caterpillars or applying their OS to wounded leaves can strongly increase the gene expression and metabolites accumulation of BXs in maize (Qi et al. 2016; Tzin et al. 2017; Guo et al. 2019; Ye et al. 2022). The induction and the toxic activity of BXs can be specific for herbivore species and developmental stage (Robert and Mateo 2022). Our results, however, suggest that OS of different *Spodoptera* species induce similar expression patterns of *BX* genes in maize leaves (Figure 6 and Supporting Information S8: Table S6). Since the BX‐dependent defences in maize leaves are highly localised (Maag et al. 2016) it should be noted that the transcriptomic analysis of whole leaf tissue in this study may dilute the BXs suppression effect of *Spodoptera* on maize and may mask the potential transcriptional differences of BX induction by different *Spodoptera* species. Also, *Spodoptera* caterpillars may manipulate maize defences through posttranscriptional regulation of BX biosynthesis and/or the transport of BXs within a plant (Maag et al. 2016), which could be the reason why we did not observe any significant suppression of *BX* gene expression upon OS application (Figure 6 and Supporting Information S8: Table S6). Mechanical damage alone was enough to induce BX biosynthesis, and this induction could be enhanced by caterpillar OS (Supporting Information S2: Figure S4A). The OS incubation assay also highlights the role of *Spodoptera* OS in inducing BX‐dependent defences in maize. The induction of BX biosynthesis seems overall stronger in *S. frugiperda* OS‐ and *S. exigua* OS‐incubated plants compared to plants incubated in *S. littoralis* OS (Supporting Information S2: Figure S4B), suggesting that the OS of *S. frugiperda* and *S. exigua* may contain higher levels of certain active chemicals that trigger BX synthesis than *S. littoralis* OS.

One of the proposed functions of HIPVs is to attract predators and parasitoids of the herbivores (Dicke and Baldwin 2010; Turlings and Erb 2018). Volatile terpenoids such as linalool (Du et al. 1998), DMNT, TMTT (Tamiru et al. 2011) and (E)‐β‐caryophyllene (Rasmann et al. 2005; Köllner et al. 2008; Xiao et al. 2012) are important components in this apparent indirect defence. TPS and some P450 monooxygenases are involved in the production and conversion of these herbivore‐induced terpenes. For example, maize TPS2 catalyzes the formation of linalool, (E)‐nerolidol and (E,E)‐geranyllinalool. Nerolidol and (E,E)‐geranyllinalool can be subsequently converted to DMNT and TMTT, respectively, by P450 monooxygenases CYP92C5 and CYP92C6 (Richter et al. 2016). Maize TPS10 catalyzes the formation of various sesquiterpenes that mediate the attractiveness of maize to parasitic wasps (Schnee et al. 2006). Maize TPS23 produces (E)‐β‐caryophyllene that attracts parasitic wasps of *S. littoralis* (Köllner et al. 2008). We found that elicitation by OS from *Spodoptera* caterpillars strongly increased the expression of four volatile‐related genes (*TPS2*, *TPS10*, *TPS23* and *CYP92C5*) in maize leaves, whereas other *TPS* genes such as *TPS4*, *TPS5* and *TPS8*, were only induced by OS incubation or by the application of a specific OS to wounds (Figure 7B). Overall, there was similarity in how different *Spodoptera* species induce the biosynthesis of HIPVs in maize and the results highlight the critical role of specific volatile‐related genes in this apparent indirect defence. Interestingly, maize plants treated with *S. frugiperda* OS always showed a less pronounced volatile emission than those treated with OS from the other two species, independent of the induction method (Figure 7C,D). This is consistent with a previous study in which herbivory by *S. frugiperda* caterpillars seems to be able to suppress the emission of HIPVs in maize when compared with more generalist lepidopteran species (De Lange et al. 2020). Since different *Spodoptera* caterpillars induced similar *TPS* gene expression patterns and no suppression of *TPS* gene expression was been detected in *S. frugiperda* infested plants (Figure 7, Supporting Information S2 and S8: Figure S5 and Table S6), we speculate that *S. frugiperda* may manipulate plant volatile emission through posttranscriptional regulation. Given the important role of stomata in indirect plant defences (Seidl‐Adams et al. 2015), *S. frugiperda* OS may also control volatile emissions in maize by manipulating stomatal aperture. Another possible explanation for why we did not observe the suppression effect of plant defence by *S. frugiperda* OS might be related to the timepoint selected for our measurements. Generally, in comparison to the responses induced by mechanical wounding alone, damage plus OS elicited significantly higher transcription of defence genes 4 h after treatment (Figure S6). OS from different *Spodoptera* species induce similar patterns of defence gene expression at almost all time points, whether it was damage plus OS treatment or OS incubation (Supporting Information S2: Figures S6 and S7, primers used for real‐time qPCR are listed in Supporting Information S9: Table S7). Interestingly, OS of *S. frugiperda* induced the lowest defence gene expression compared to the other two *Spodoptera* species only at the earliest timepoint (0.5 h) (Supporting Information S2: Figure S6). Although this trend was not statistically significant for all genes, it might still suggest that OS of *S. frugiperda* contains effectors that suppress the defence response at a very early stage of OS application. Alternatively, effectors in *S. frugiperda* OS have little bioactivity in vitro, but during actual larval feeding, *S. frugiperda* may effectively suppress maize defence responses by continuously depositing active effectors (De Lange et al. 2020). The mechanism underlying the possible manipulation by *S. frugiperda* of maize volatile emissions remains to be determined.

In this study, we evaluated the changes in the transcriptome, phytohormones, and volatile emissions in maize plants upon elicitation by OS from three *Spodoptera* species (Supporting Information S2: Figure S8). As most measurements were done at a single temporal snapshot after plant treatment, future time‐course analyses will be needed to provide further details on the dynamics of these responses. Still, the current results provide valuable insights into the specificity and generality of orchestrated defence responses in maize against these closely related herbivores and provide a basis for the identification of herbivore‐specific elicitors/effectors, paving the way for novel crop protection strategies.

## Conflicts of Interest

The authors declare no conflicts of interest.

## Supporting information


**Data S1:** Genes detected in all samples. **Data S2:** Differentially expressed genes (DEGs) in maize leaves in response to different treatments with a cut‐off of two‐fold change relative to specific controls. **Data S3:** Differentially expressed genes (DEGs) specifically induced by oral secretions from different *Spodoptera* caterpillars. **Data S4:** Differentially expressed genes (DEGs) in maize leaves in response to different treatments with a cut‐off of two‐fold change relative to the unmanipulated controls.

**Figure S1:** Differentially expressed genes (DEGs) in maize leaves in response to treatments with oral secretion (OS) of different *Spodoptera* species compared with unmanipulated controls. **Figure S2:** KEGG (Kyoto Encyclopedia of Genes and Genomes) pathway enrichment analysis of differentially expressed genes (DEGs) in maize induced by different treatments compared with corresponding control for mechanical damage plus oral secretion (OS) application and OS incubation experiment. **Figure S3:** Effects of treatment with oral secretion (OS) of different *Spodoptera* species on gene expression involved in ethylene biosynthesis pathway. **Figure S4:** Mean transcript levels of benzoxazinoid (BX) biosynthetic genes in maize plants after treatments of mechanical damage plus OS application experiment or OS incubation experiment. **Figure S5:** Mean transcript levels of volatile terpene biosynthetic genes in maize plants after treatments of mechanical damage plus OS application experiment or OS incubation experiment. **Figure S6:** The transcript levels of four genes in leaves of maize seedlings at different time points after treatments of mechanical damage plus OS application experiment. **Figure S7:** The transcript levels of eight genes in leaves of maize seedlings at different time points after treatments of OS incubation experiment. **Figure S8:** Summary of the integrated multi‐omics analysis of maize perception and response to oral secretion (OS) of three closely related *Spodoptera* species.

**Table S1:** Summary of RNA sequencing and mapping using the maize genome as the reference.

**Table S2:** Summary statistics for Figure 2.

**Table S3:** Gene descriptions of top and bottom loadings of first principal component (PC1) and second principal component (PC2).

**Table S4:** KEGG pathway enrichment analysis of DEGs between each treatment and the specific control.

**Table S5:** The gene expression pattern of phytohormones.

**Table S6:** The gene expression pattern of benzoxazinoids and volatile terpenes.

**Table S7:** Primers used for qRT‐PCR.

## Data Availability

The data that supports the findings of this study are available in the Supporting Information material of this article.
