# Supplementary material for: Three Closely Related Spodoptera Species Similarly Affect Gene Expression and Phytohormone Levels but Differentially Induce Volatile Emissions in Maize
Source: Plant Cell Environ. 2026 Jan 19;49(5):2394–412. doi: 10.1111/pce.70389 (PMC13051760; doi:10.1111/pce.70389)
Supplement: Supplementary file 2 — Figure S1: Differentially expressed genes (DEGs) in maize leaves in response to treatments with oral secretion (OS) of different Spodoptera species compared with unmanipulated controls. Figure S2: KEGG (Kyoto Encyclopedia of Genes and Genomes) pathway enrichment analysis of differentially expressed genes (DEGs) in maize induced by different treatments compared with corresponding control for mechanical damage plus oral secretion (OS) application and OS incubation experiment. Figure S3: Effects of treatment with oral secretion (OS) of different Spodoptera species on gene expression involved in ethylene biosynthesis pathway. Figure S4: Mean transcript levels of benzoxazinoid (BX) biosynthetic genes in maize plants after treatments of mechanical damage plus OS application experiment or OS incubation experiment. Figure S5: Mean transcript levels of volatile terpene biosynthetic genes in maize plants after treatments of mechanical damage plus OS application experiment or OS incubation experiment. Figure S6: The transcript levels of four genes in leaves of maize seedlings at different time points after treatments of mechanical damage plus OS application experiment. Figure S7: The transcript levels of eight genes in leaves of maize seedlings at different time points after treatments of OS incubation experiment. Figure S8: Summary of the integrated multi‐omics analysis of maize perception and response to oral secretion (OS) of three closely related Spodoptera species. [file PCE-49-2394-s005.docx]

**Three closely related *Spodoptera* species similarly affect gene expression and phytohormone levels but differentially induce volatile emissions in maize**

Wenfeng Ye^‡,1,2^, Sara Leite Dias^‡,1,3^, Marine Mamin^1^, Carla C.M. Arce^1^, Ted C.J. Turlings^1,*^

**Supporting information**

**Supplemental experimental procedures**

**Principal component analysis (PCA) and powered partial least squares–discriminant analysis (PPLS-DA)**

Principal component analysis (PCA) was performed using PCAexplorer (Marini and Binder, 2019). The analysis used variance stabilized transformed data as input, and the 10,000 of most variant genes were selected for PCA. Genes in the top and bottom loadings of the first and second principal component were listed in Table S3. Powered partial least squares–discriminant analysis (PPLS-DA) was carried out using the cppls function from the pls package. Model performance was evaluated by estimating the classification error rate via cross-model validation with the MVA.cmv function. The statistical significance of discrimination was assessed using a permutation test in the MVA.test function. Both MVA.cmv and MVA.test are from the RVAideMemoire package.

**Total RNA isolation and Real-time qPCR**

Leaf samples were harvested at different time points after the start of the treatment in mechanical damage plus OS application experiment (MD+OS) and OS incubation experiment. Frozen leaves were ground into a fine powder in a mortar filled with liquid nitrogen. Total RNA was extracted using the GeneJET Plant Purification Mini Kit (Thermo Fisher Scientific Baltics UAB, Vilnius, Lithuania) according to the manufacturer’s instructions and DNA removal was performed using the RNase-Free DNase Set (QIAGEN, Hilden, Germany). Each RNA sample (500 ng) was reverse transcribed using the GoScript™ Reverse Transcription System (Promega). Real-time qPCR was performed on the Rotor-Gene™ 6000 (Corbett Research) using GoTaq® qPCR Master Mix (Promega). Primers used for real-time qPCR are listed in Table S7. For the expression analysis of each gene in MD+OS experiment, samples from uninfested control maize plants were designated as calibrator. Each treatment and time point was replicated four times. For the expression analysis of each gene in OS incubation experiment, samples from cut maize leaves incubated in distilled water (I-W) were designated as calibrator. Each treatment and time point was replicated three times. Relative expression levels of each gene were normalized with the *ZmCUL* gene (Cullin, locus tag: Zm00001d024855) (Manoli et al., 2012) and calculated using the 2^-△△Ct^ method (Livak and Schmittgen, 2001).


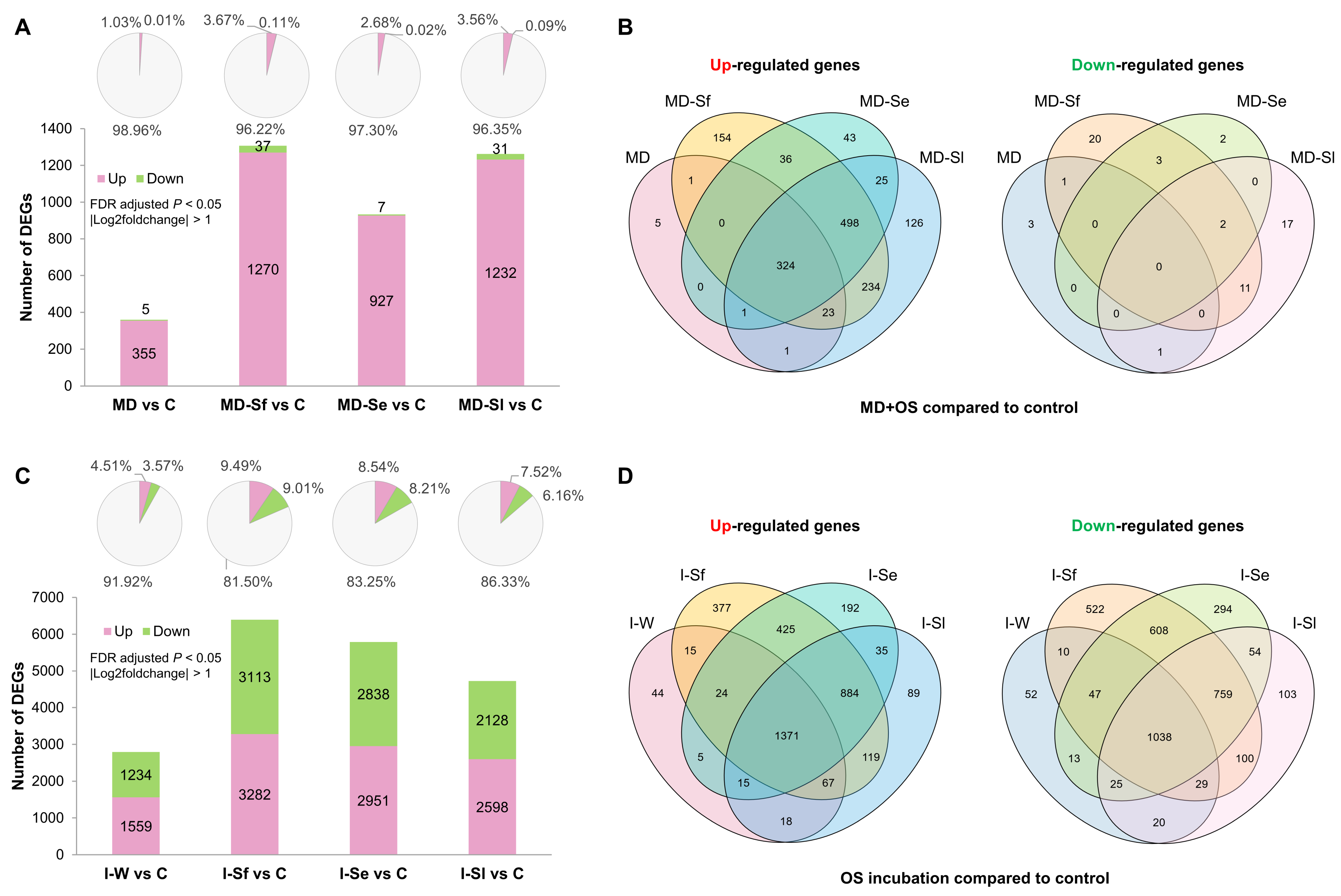


**Figure S1**. Differentially expressed genes (DEGs) in maize leaves in response to treatments with oral secretion (OS) of different *Spodoptera* species compared with unmanipulated controls. (**A**) Total number of DEGs that were significantly upregulated or downregulated in maize leaves in response to mechanical leaf damage (MD), or mechanical damage plus application of OS from *S. frugiperda* (MD-Sf), *S. exigua* (MD-Se), or *S. littoralis* (MD-Sl) compared with unmanipulated controls (C). The pie chart indicates the percentage of DEGs respectively regulated by MD, MD-Sf, MD-Se, and MD-Sl for all 34,567 present genes across 27 cDNA libraries. (**B**) Venn diagram illustrating the number of specifically and commonly upregulated and downregulated DEGs in maize in response to MD, MD-Sf, MD-Se, or MD-Sl compared with controls. (**C**) Total number of DEGs that were significantly upregulated or downregulated in maize leaves after water incubation (I-W) or incubation in the OS of *S. frugiperda* (I-Sf), *S. exigua* (I-Se), or *S. littoralis* (I-Sl) compared with unmanipulated controls (C). The pie chart indicates the percentage of DEGs respectively regulated by I-W, I-Sf, I-Se, and I-Sl for all 34,567 present genes across 27 cDNA libraries. (**D**) Venn diagram illustrating the number of specifically and commonly regulated DEGs in maize in response to I-W, I-Sf, I-Se, or I-Sl.


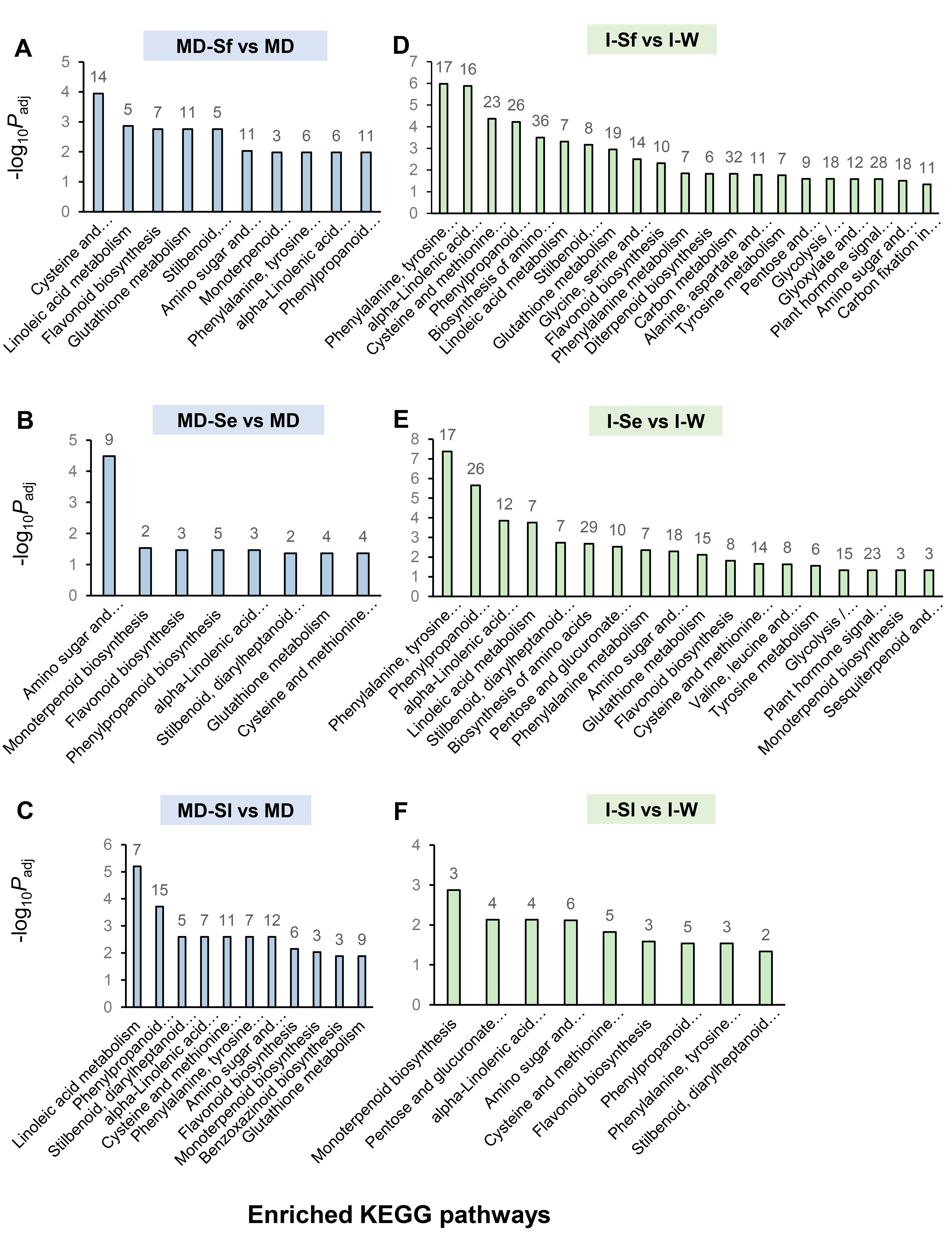


**Figure S2**. KEGG (Kyoto Encyclopedia of Genes and Genomes) pathway enrichment analysis of differentially expressed genes (DEGs) in maize induced by different treatments compared with corresponding control in mechanical damage plus oral secretion (OS) application and OS incubation experiment. (**A, B, and C**) KEGG pathways of DEGs in maize between mechanical damage plus application of OS of *S. frugiperda* (MD-Sf), *S. exigua* (MD-Se), *S. littoralis* (MD-Sl), and mechanical damage alone (MD). (**D, E, and F**) KEGG pathways of DEGs in maize between incubation in OS of *S.* *frugiperda* (I-Sf), *S. exigua* (I-Se), *S. littoralis* (I-Sl), and water incubation (I-W). Enrichment scores are shown as -log_10_(adjusted *P* value). Number of DEGs involved in each term are shown above the bar. For full datasets, refer to Table S4.


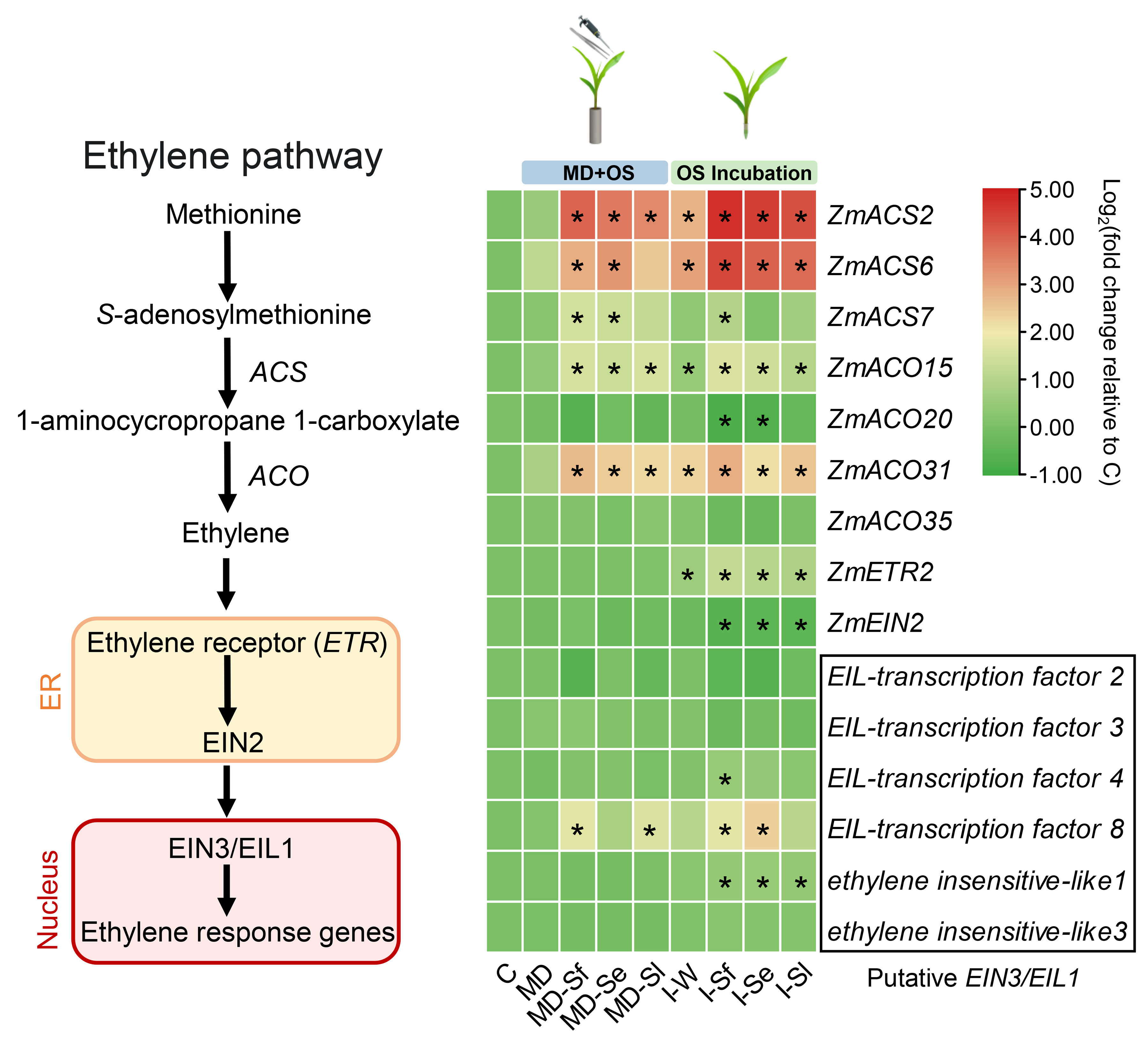


**Figure S3**. Effects of treatment with oral secretion (OS) of different *Spodoptera* species on gene expression involved in ethylene biosynthesis pathway. Plants were subjected to the following treatments in the mechanical damage plus OS application experiment (MD+OS): mechanically damaged (MD), mechanically damaged and application of OS of *Spodoptera frugiperda* (MD-Sf), *Spodoptera exigua* (MD-Se), or *Spodoptera littoralis* (MD-Sl). Treatments in the OS incubation experiment: incubation of cut maize leaves in distilled water (I-W) or in 10% OS of *S. frugiperda* (I-Sf), *S. exigua* (I-Se), or *S. littoralis* (I-Sl). Control plants (C) were kept untreated. Schematic diagram and heat map depict the biosynthesis pathway and the gene expression pattern of ethylene pathway. ER, endoplasmic reticulum. Color coding represents the range of log_2_(fold change relative to control). Genes differentially expressed between control and treatments are indicated by stars (false discovery rate (FDR) adjusted *P* < 0.05). For full datasets, refer to Table S5.


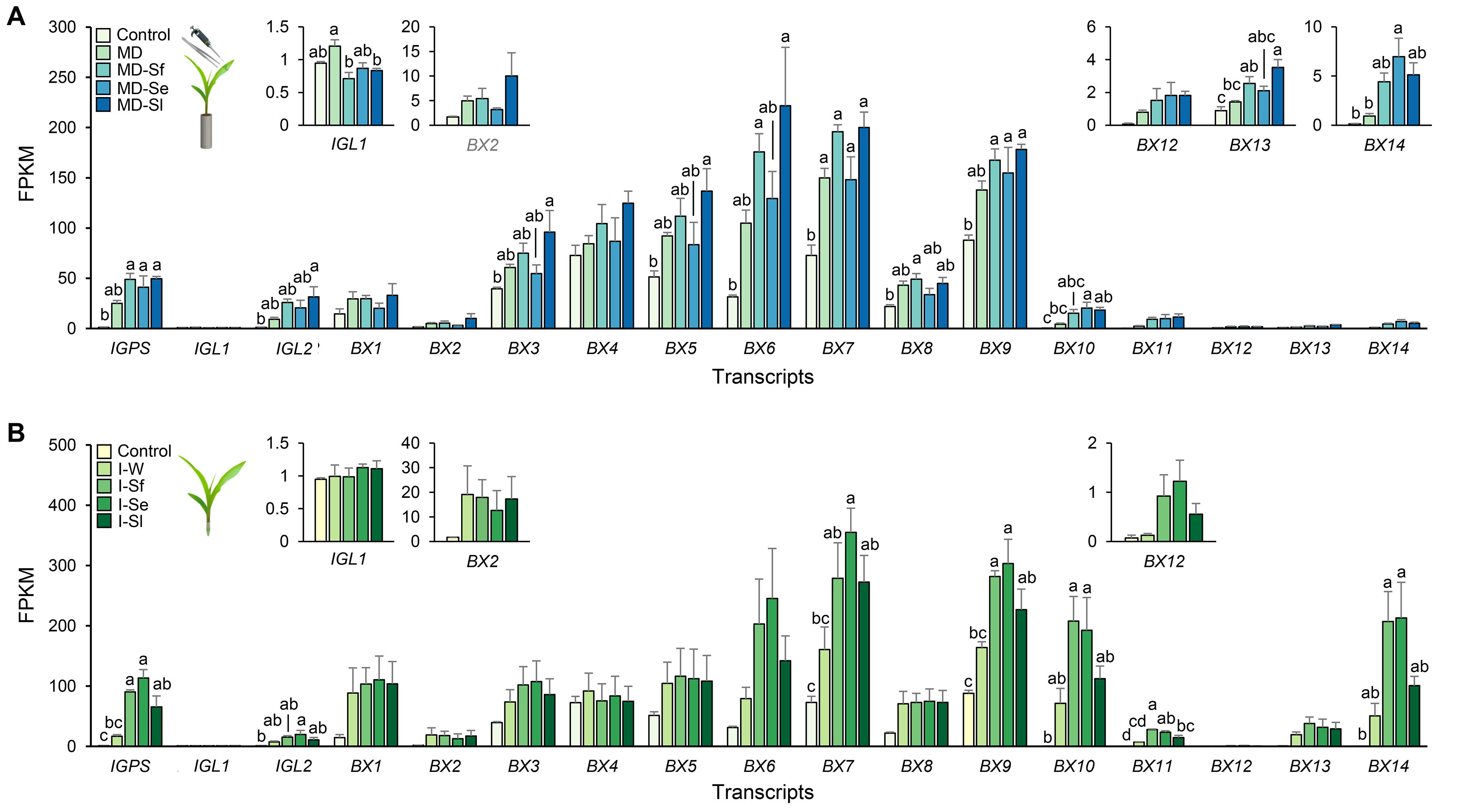


**Figure S4**. Mean transcript levels (+SE, *n* = 3) of benzoxazinoid (BX) biosynthetic genes in maize plants after treatments of mechanical damage plus OS application experiment (MD+OS) (**A**) or OS incubation experiment (**B**). Plants were subjected to the following treatments in the MD+OS experiment: mechanically damaged (MD), mechanically damaged and application of OS of *Spodoptera frugiperda* (MD-Sf), *Spodoptera exigua* (MD-Se), or *Spodoptera littoralis* (MD-Sl). Treatments in the OS incubation experiment: incubation of cut maize leaves in distilled water (I-W) or in 10% OS of *S. frugiperda* (I-Sf), *S. exigua* (I-Se), or *S. littoralis* (I-Sl). Control plants were kept untreated. FPKM, fragments per kilobase of transcript per million fragments mapped. Different letters indicate significant differences among treatments for each gene (ANOVA, pairwise comparisons of EMMeans, *P* < 0.05). For full datasets, refer to Table S6.


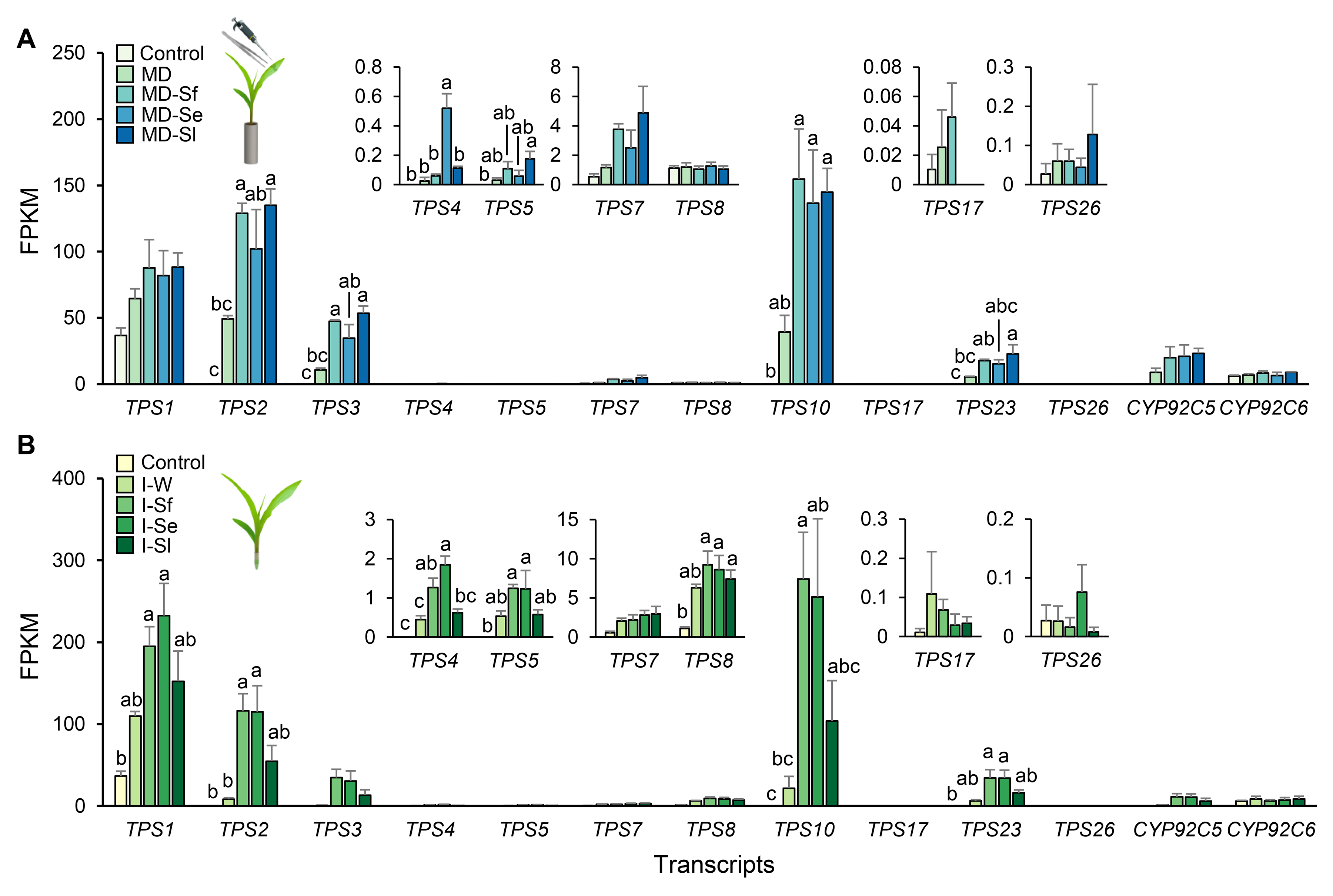


**Figure S5**. Mean transcript levels (+SE, *n* = 3) of volatile terpene biosynthetic genes in maize plants after treatments of mechanical damage plus OS application experiment (MD+OS) (**A**) or OS incubation experiment (**B**). Plants were subjected to the following treatments in the MD+OS experiment: mechanically damaged (MD), mechanically damaged and application of OS of *Spodoptera frugiperda* (MD-Sf), *Spodoptera exigua* (MD-Se), or *Spodoptera littoralis* (MD-Sl). Treatments in the OS incubation experiment: incubation of cut maize leaves in distilled water (I-W) or in 10% OS of *S. frugiperda* (I-Sf), *S. exigua* (I-Se), or *S. littoralis* (I-Sl). Control plants were kept non-manipulated. FPKM, fragments per kilobase of transcript per million fragments mapped. Different letters indicate significant differences among treatments for each gene (ANOVA, pairwise comparisons of EMMeans, *P* < 0.05). For full datasets, refer to Table S6.


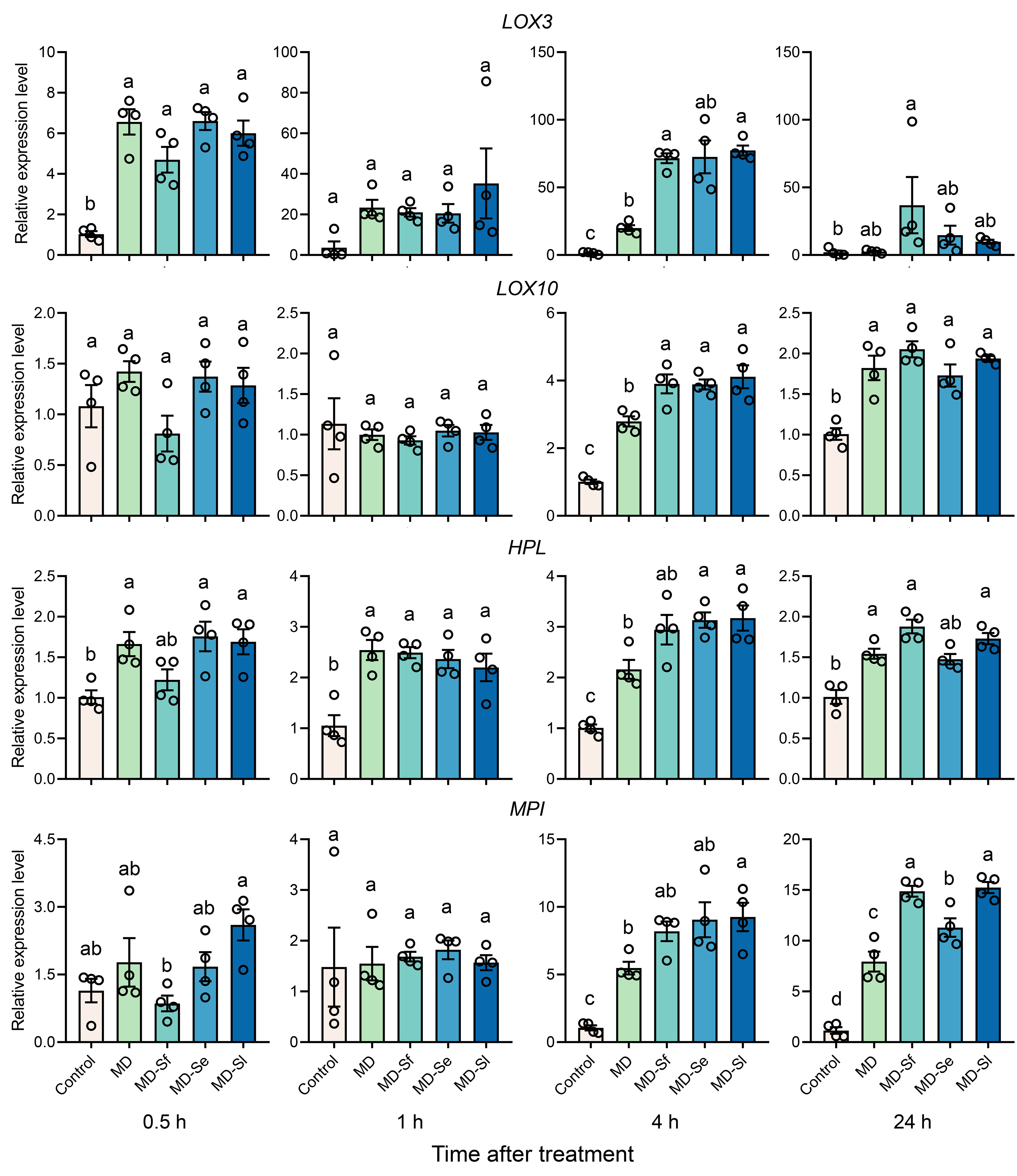


**Figure S6**. The transcript levels (mean + SE, *n* = 4) of four genes in leaves of maize seedlings at different time points after treatments of mechanical damage plus OS application experiment (MD+OS). Plants were subjected to the following treatments: mechanically damaged (MD), mechanically damaged and application of OS of *Spodoptera frugiperda* (MD-Sf), *Spodoptera exigua* (MD-Se), or *Spodoptera littoralis* (MD-Sl). Control plants were kept non-manipulated. The following genes were measured: *LOX3* (lipoxygenase, Zm00001d033623), *LOX10* (lipoxygenase, Zm00001d053675), *HPL* (hydroperoxide lyase, Zm00001d054067), and *MPI* (maize protease inhibitor, Zm00001d011080). Fold-change of gene expression level was calculated using the 2^-ΔΔCT^ method. The results (threshold cycle values) of the RT-PCR assays were normalized to the expression level of *ZmCUL* (cullin, Zm00001d024855).


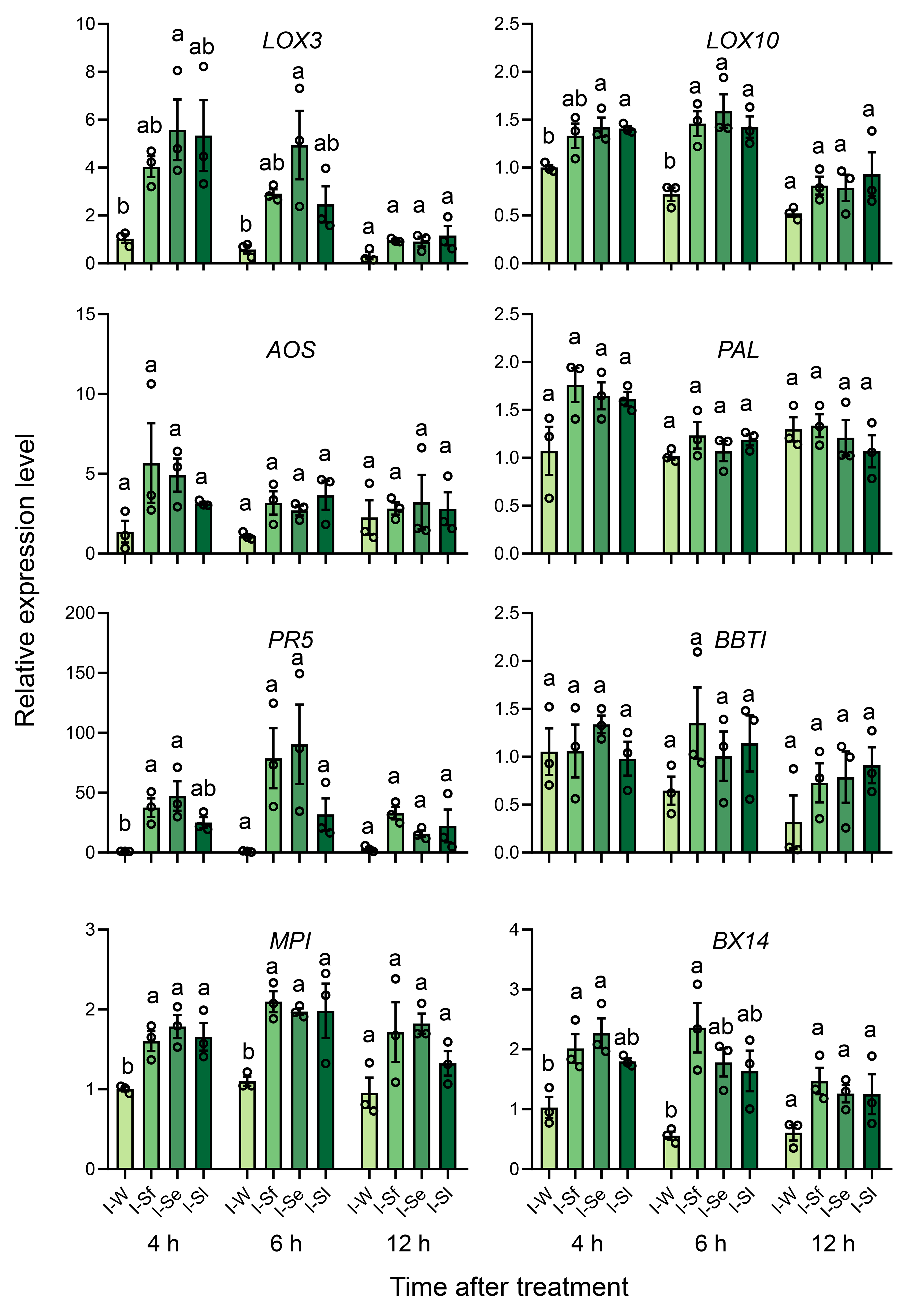


**Figure S7**. The transcript levels (mean + SE, *n* = 3) of eight genes in leaves of maize seedlings at different time points after treatments of OS incubation experiment. Plants were subjected to the following treatments: incubation of cut maize leaves in distilled water (I-W) or in 10% OS of *S. frugiperda* (I-Sf), *S. exigua* (I-Se), or *S. littoralis* (I-Sl). The following genes were measured: *LOX3* (lipoxygenase, Zm00001d033623), *LOX10* (lipoxygenase, Zm00001d053675), *AOS* (allene-oxide synthase 4, Zm00001d034184), *PAL* (phenylalanine ammonia lyase 9, Zm00001d017275), *PR5* (pathogenesis-related protein 5, Zm00001d031158), *BBTI* (Bowman-Birk type trypsin inhibitor, Zm00001d048660), *MPI* (maize protease inhibitor, Zm00001d011080), and *BX14* (benzoxazinone synthesis 14, Zm00001d004921). Fold-change of gene expression level was calculated using the 2^-ΔΔCT^ method. The results (threshold cycle values) of the RT-PCR assays were normalized to the expression level of *ZmCUL* (cullin, Zm00001d024855).


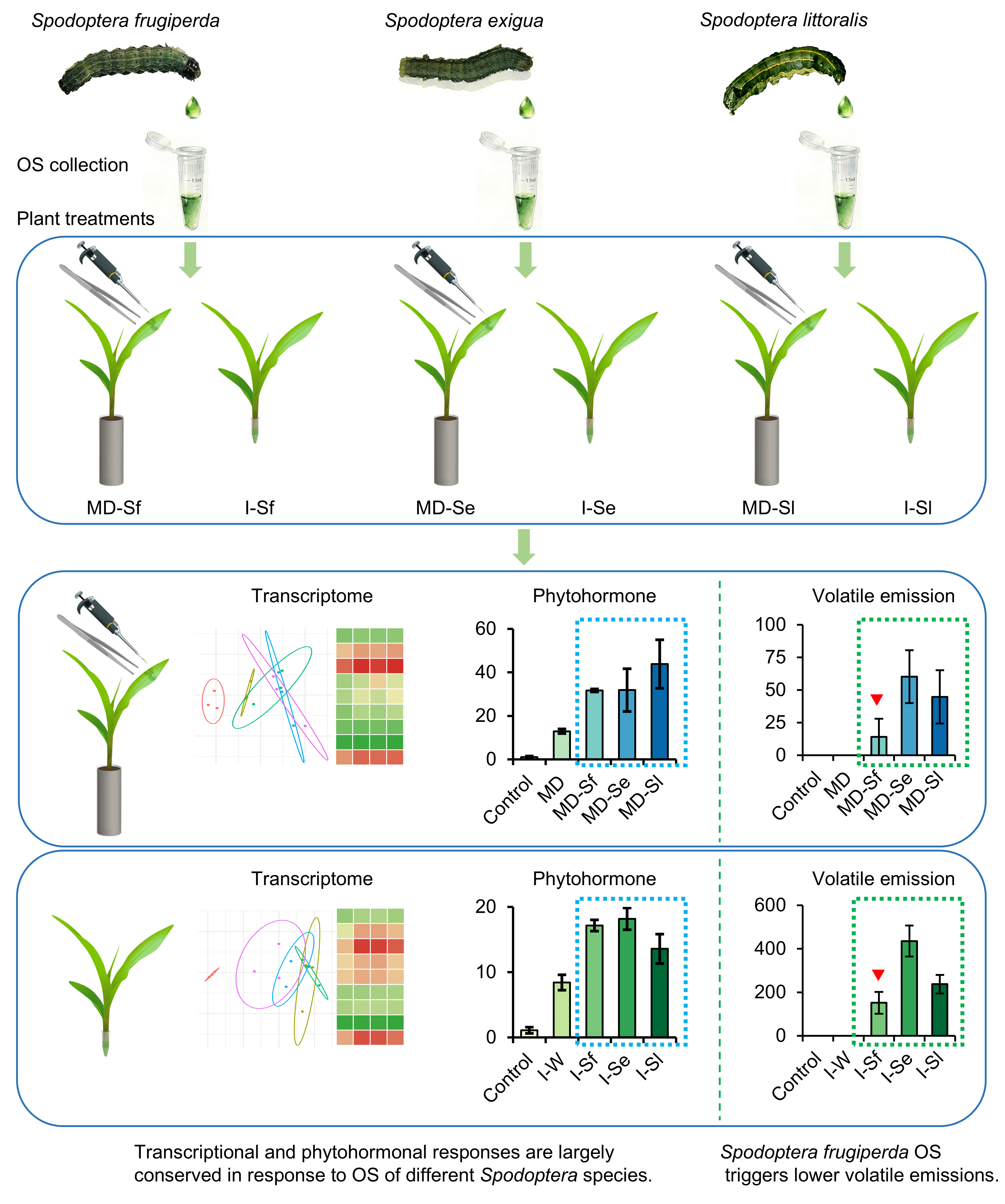


**Figure S8**. Summary of the integrated multi-omics analysis of maize perception and response to oral secretion (OS) of three closely related *Spodoptera* species.

**Data S1 to S4 and Table S1 to S6 are attached in separate documents**

**Data S1** Genes detected in all samples.

**Data S2** Differentially expressed genes (DEGs) in maize leaves in response to different treatments with a cut-off of two-fold change relative to specific controls.

**Data S3** Differentially expressed genes (DEGs) specifically induced by oral secretions from different *Spodoptera* caterpillars.

**Data S4** Differentially expressed genes (DEGs) in maize leaves in response to different treatments with a cut-off of two-fold change relative to the unmanipulated controls.

**Table S1** Summary of RNA sequencing and mapping using the maize genome as the reference.

**Table S2** Summary statistics for Figure 2.

**Table S3** Gene descriptions of top and bottom loadings of first principal component (PC1) and second principal component (PC2).

**Table S4** KEGG pathway enrichment analysis of DEGs between each treatment and the specific control.

**Table S5** The gene expression pattern of phytohormones.

**Table S6** The gene expression pattern of benzoxazinoids and volatile terpenes.

**Table S7** Primers used for qRT-PCR.

**Supplemental references**

**Marini F. and Binder H.** (2019). pcaExplorer: an R/Bioconductor package for interacting with RNA-seq principal components. ***BMC Bioinformatics*** **20**:331. https://doi.org/10.1186/s12859-019-2879-1

**Manoli, A., Sturaro, A., Trevisan, S., Quaggiotti, S., and Nonis, A.** (2012). Evaluation of candidate reference genes for qPCR in maize. *J. Plant Physiol.* **169**:807–815. https://doi.org/10.1016/j.jplph.2012.01.019

**Livak, K. J., and Schmittgen, T. D.** (2001). Analysis of relative gene expression data using real-time quantitative PCR and the 2^-ΔΔCT^ method. *Methods* **25**:402–408. https://doi.org/10.1006/meth.2001.1262
